# Adaptive laboratory evolution of β-caryophyllene producing *Saccharomyces cerevisiae*

**DOI:** 10.1186/s12934-021-01598-z

**Published:** 2021-05-27

**Authors:** Avinash Godara, Katy C. Kao

**Affiliations:** 1 Department of Chemical Engineering, Texas A&M University, College Station, TX USA; 2 Department of Chemical and Materials Engineering, San Jose State University, One Washington Sq, San Jose, CA 95192 USA

**Keywords:** Adaptive laboratory evolution, β-Caryophyllene, Selective pressure, *Saccharomyces cerevisiae*, CRISPR–Cas9

## Abstract

**Background:**

β-Caryophyllene is a plant terpenoid with therapeutic and biofuel properties. Production of terpenoids through microbial cells is a potentially sustainable alternative for production. Adaptive laboratory evolution is a complementary technique to metabolic engineering for strain improvement, if the product-of-interest is coupled with growth. Here we use a combination of pathway engineering and adaptive laboratory evolution to improve the production of β-caryophyllene, an extracellular product, by leveraging the antioxidant potential of the compound.

**Results:**

Using oxidative stress as selective pressure, we developed an adaptive laboratory evolution that worked to evolve an engineered β-caryophyllene producing yeast strain for improved production within a few generations. This strategy resulted in fourfold increase in production in isolated mutants. Further increasing the flux to β-caryophyllene in the best evolved mutant achieved a titer of 104.7 ± 6.2 mg/L product. Genomic analysis revealed a gain-of-function mutation in the a-factor exporter *STE6* was identified to be involved in significantly increased production, likely as a result of increased product export.

**Conclusion:**

An optimized selection strategy based on oxidative stress was developed to improve the production of the extracellular product β-caryophyllene in an engineered yeast strain. Application of the selection strategy in adaptive laboratory evolution resulted in mutants with significantly increased production and identification of novel responsible mutations.

**Supplementary Information:**

The online version contains supplementary material available at 10.1186/s12934-021-01598-z.

## Background

Terpenoids are the largest and most diverse family of plant-derived compounds found in nature. They are high-value compounds with wide industrial applications ranging from fuel alternatives, nutraceuticals, pharmaceutics, etc. Terpenes are secondary metabolites that are derived from C_5_ precursor isopentenyl diphosphate (IPP) or dimethylallyl diphosphate (DMAPP) [1, 2]. Extracting them from their natural source is usually not economical because of their low abundance. Thus, microbial-based biosynthesis of terpenoids has been explored as a sustainable alternative for industrial production [3–5].

Sesquiterpene is a family of terpenoids containing three isoprene units and they can be monocyclic, bicyclic or tricyclic in structure [6, 7]. β-caryophyllene is a bicyclic sesquiterpene and has antioxidant and anti-inflammatory properties, with potential applications as an aircraft fuel alternative and a potential therapeutic compound due to its antioxidant and anti-inflammatory properties [8, 9]. Microbial production of β-caryophyllene has been demonstrated in *E. coli* and cyanobacteria reaching titers of ~ 1.5 g/L in a fed-batch bioreactor and 46.6 μg/L, respectively [10, 11].

Apart from rationally engineering the known terpenoid biosynthesis pathway, due to complex and interlinked nature of metabolic network and cellular physiology, genes and pathways not directly connected to the biosynthetic pathway may influence product formation [12, 13]. A clear knowledge of the genotype–phenotype relationship for product formation is not fully known. Thus, complementary techniques to rational engineering such as screening deletion or overexpression libraries [14–16] and adaptive laboratory evolution (ALE)-based strategies [17, 18] can help to identify additional gene and/or pathway targets related with increasing product formation. In recent work, Promdonkoy et al. utilized both rational engineering and ALE to improve the d-xylose utilization and isobutanol production in *Saccharomyces cerevisiae* [19]. In another work, Rugbjerg et al. showed evolved *Escherichia coli* MG1655 which utilizes glucose more efficiently due to a mutation in *rpoB* also exhibited improved mevalonate productivity [20].

Previously, we developed an environmental engineering-based strategy using ALE to improve product formation of an intracellular product with antioxidant properties by applying periodic oxidative stress challenge [18]. With extracellular products, the same strategy may fail due to potential “cheating” by non-producers in the population. In this work, we explore the potential application of ALE for the production of an extracellular product β-caryophyllene in *S. cerevisiae*. Initial pathway engineering led to a strain producing ~ 3.8 mg/g DCW β-caryophyllene. The optimized strain was used to design an optimum oxidative stress challenge strategy to improve product formation using ALE. Using the optimized strategy, evolved mutants that exhibit a fourfold increase in β-caryophyllene biosynthesis were isolated and characterized. Subsequent genotypic analyses led to the identification of two beneficial mutations responsible for the enhanced production phenotype observed.

## Results and discussion

### Optimization of oxidative stress for use in adaptive laboratory evolution

Selecting an appropriate selective pressure (stressor) is key to successfully use of adaptive laboratory evolution. Since β-caryophyllene is a known antioxidant [9, 21], an oxidative stress-based selective pressure can be used to aid the coupling of cellular growth or survival with production [18]. The strategy is based on the hypothesis that in an oxidative environment, the strain that produces more of an antioxidant product will have a growth advantage. To determine whether the production of β-caryophyllene increased cell survival, the *QHS1* gene encoding β-caryophyllene synthase from *Artemisia annua*, which has been successfully used to biosynthesize β-caryophyllene in *E. coli* [11] was codon optimized for yeast and expressed in strain BY4741. The catalase gene encoded by *CTT1* was deleted to reduce the yeast native defense against hydrogen peroxide. Then strains that produce varying levels of β-caryophyllene were constructed in the *ctt1*∆ strain, resulting in non-producers YAG110 (BY4741 *ctt1*Δ) and YAG114 (YAG110 with a genome-integrated farnesyl pyrophosphate [FPP] overproduction cassette), and producers YAG111 (YAG110 with the β-caryophyllene synthase *QHS1* gene integrated in the genome) and YAG115 (YAG110 with *QHS1* and FPP overproduction cassette integrated in the genome). See Additional file 1: Figure S1 and “Materials and methods” section for description of genes used in metabolic engineering. Initial experiment was conducted to measure the level of β-caryophyllene accumulated intracellularly and in the culture. No β-caryophyllene was detected from the intracellular measurements (data not shown), suggesting that this is an extracellular product. Production results showed YAG115 producing higher amount of β-caryophyllene than YAG111 (Additional file 1: Table S1). All four strains were challenged with different concentrations of H_2_O_2_ (0 mM, 50 mM, 100 mM, 150 mM and 200 mM) and their relative viabilities were assessed (results are shown in Additional file 1: Figure S2). The non-producers YAG110 and YAG114 showed the lowest levels of tolerance with ~ 10% survival with 50 mM H_2_O_2_. Strain YAG111 showed an intermediate level of oxidative stress tolerance with ~ 10% survival after challenge with 150 mM H_2_O_2_. The strain with the highest level of β-caryophyllene production YAG115 exhibited the highest tolerance with ~ 10% survival at 200 mM H_2_O_2_.

In addition to survival after 30 min challenge with higher concentrations of H_2_O_2_, cellular growth during continuous exposure at growth-permissible concentrations were also evaluated for each strain. H_2_O_2_ concentrations ranging from 0 mM, 25 mM, 50 mM, 75 mM and 100 mM were directly added to the media, and growth kinetics were measured. The data (in Additional file 1: Table S2) showed no growth in the non-producer YAG110 for any H_2_O_2_ concentrations above 0 mM. Strain YAG111 and YAG114 showed some growth in 100 mM and 50 mM respectively. The higher tolerance of YAG114 over YAG110 may be attributed to increased accumulation of sterol because of the presence of the FPP overproduction cassette. The highest producer, YAG115, was the only strain able to grow in 100 mM hydrogen peroxide, although with a very long lag phase and with a low final cell density. This data showed the possibility of using continuous exposure as a possible alternative to periodic challenge as environmental stress for ALE experiment.

**Fig. 1:**
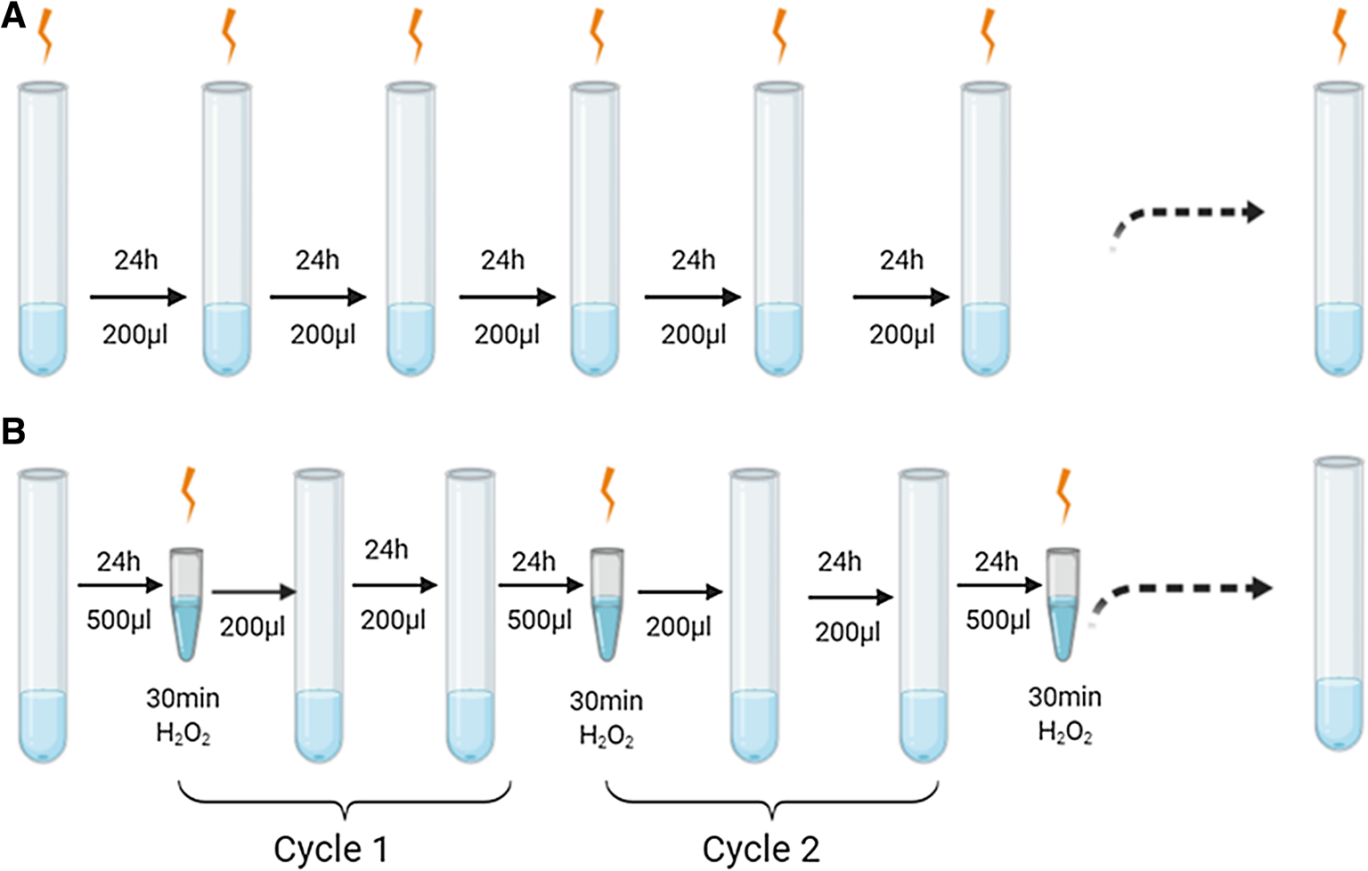
Schematic of different stress strategy tested. **A** Continuous exposure with H_2_O_2_ directly added to media. **B** Periodic exposure to H_2_O_2_ for 30 min, followed by a recovery period

**Fig. 2:**
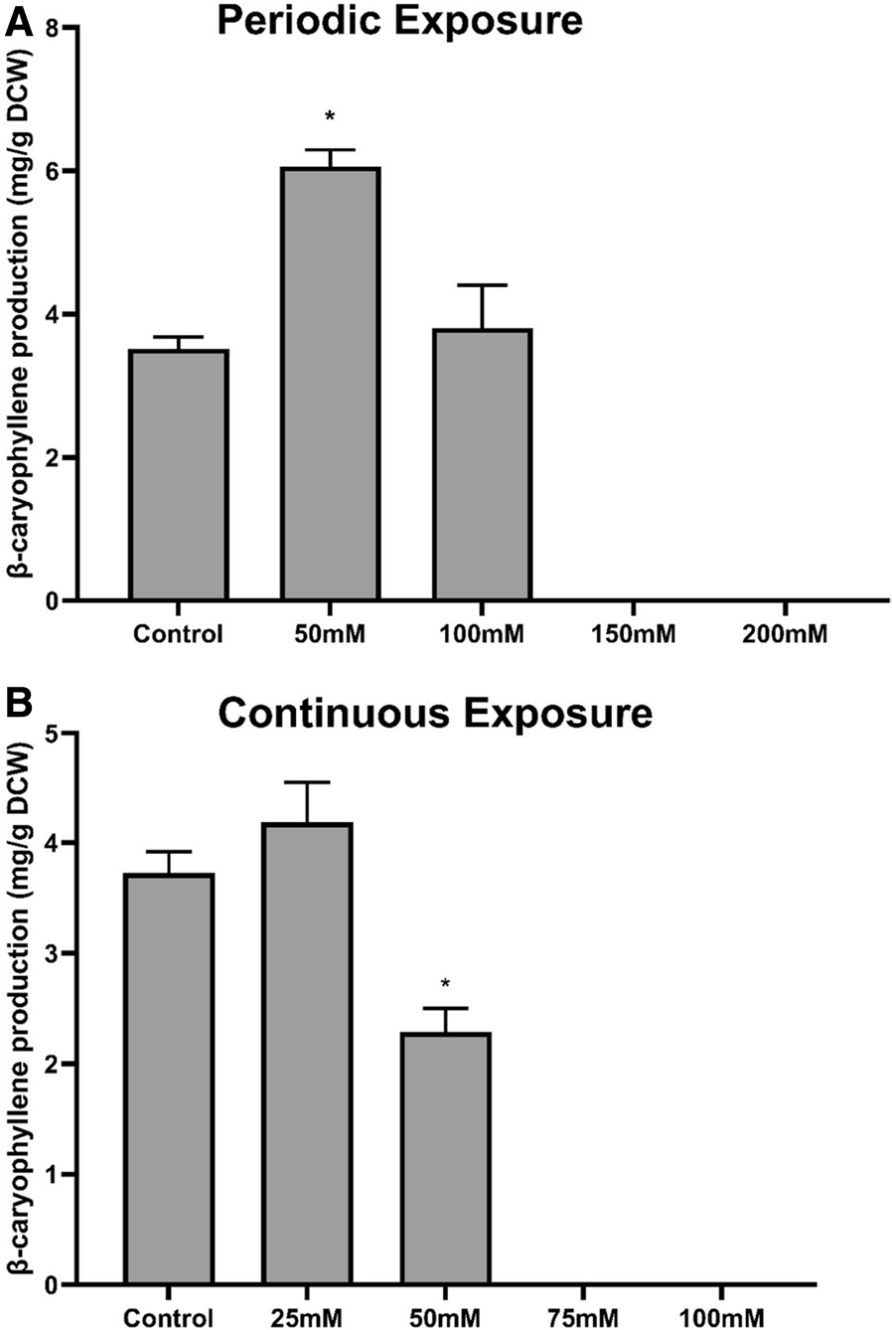
Average β-caryophyllene production observed after short-term selection using hydrogen peroxide for 8 days using **A** periodic challenge and **B** continuous exposure at various hydrogen peroxide concentrations. Production was measured in 20 mL test tube with 3 mL culture overlayed with 500 μL dodecane. Asterisks: p value < 0.05 using two-tailed Student’s t-test against control

YAG115 was chosen as the parental strain for ALE to improve β-caryophyllene production since this strain exhibited the highest tolerance to oxidative stress. Prior to initiating an evolution experiment, two types of oxidative stress selection strategies were evaluated for their ability to improve β-caryophyllene production. A continuous exposure strategy, where the cultures are continuously exposed to growth-permissible H_2_O_2_ in the media, and a periodic exposure strategy, where the cultures are subjected to a 30 min H_2_O_2_ challenge followed by a recovery period as described in Fig. 1, were used. In all cases, a dodecane layer (500 µL) was added to each 3 mL of culture for β-caryophyllene capture. After every 24 h of growth, ~ 7% (200 μL) of the culture was used to inoculate the subsequent culture (3 mL of fresh media). Each strategy was evaluated for β-caryophyllene titer and yield after 8 days (continuous strategy) or 4 cycles (periodic challenge strategy). Results showed no significant increase in yield using continuous exposure at 25 mM H_2_O_2_ compared to the no stressor control (Fig. 2). A decrease in total yield was observed for all other H_2_O_2_ concentrations using continuous exposure. On the other hand, significant increases of up to 1.7-fold in production were observed in populations subjected to periodic challenge with 50 mM H_2_O_2_. Periodic challenge with 100 mM H_2_O_2_ resulted in an insignificant increase in production, with higher concentrations showing complete loss of production. The results revealed that periodic challenge at a H_2_O_2_ concentration that resulted in ~ 10% survival was an optimal selection strategy for increasing β-caryophyllene production using ALE.

### Increased β-caryophyllene production via adaptive laboratory evolution using periodic H_2_O_2_ challenge

An ALE experiment was initiated with YAG115 using the periodic challenge strategy with an initial concentration of 50 mM H_2_O_2_. To further optimize the ramp-up in the ALE, at the end of cycle 2 (day 5), the culture was split into two populations, P1 and P2, which were exposed to 50 mM and 100 mM H_2_O_2_ exposure, respectively, in ramping up the selection pressure (Additional file 1: Figure S3). The next split and ramp-up was done at the end of cycle 4 (day 9) from the P2 population (100 mM exposure) into P3 and P4, which were subsequently exposed to 150 mM and 200 mM H_2_O_2_ exposure, respectively. After the split, four populations (P1, P2, P3, and P4) were maintained. At cycle 5, P1 and P2 reached a peak in production, reaching ~ threefold increase in β-caryophyllene production before a stagnation or decrease in production were observed. On the other hand, populations P3 and P4 showed consistent decreases in production over time. Thus, population P2 was chosen for further analyses.

### Isolated evolved mutants exhibit significant increase in production

Under the assumption that evolved mutants with higher production of β-caryophyllene should have enhanced survival after oxidative stress challenge compared to the parental strain, we first identified a concentration of H_2_O_2_ in which the parental strain has negligible viability after a 30-min exposure. As shown in Additional file 1: Figure S4, 1 M H_2_O_2_ exposure resulted in no growth of the parental strain, whereas evolved population P2 (day 11) had ~ 10% viability. In addition, we hypothesized that colony size after H_2_O_2_ challenge can be used to estimate relative production. Population P2 (day 11) was exposed to 1 M hydrogen peroxide stress for 30 min and plated on SC-ura-leu plate. Three sizes of colonies were observed, small, medium and large. These colonies were cultured and their β-caryophyllene production were quantified. A positive correlation was observed in size and production as medium and large colonies (see Additional file 1: Figure S5). The result suggests a growth advantage of mutants with higher production of β-caryophyllene, indicating the evolution strategy resulted in growth/survival and production coupling as hypothesized.

**Fig. 3:**
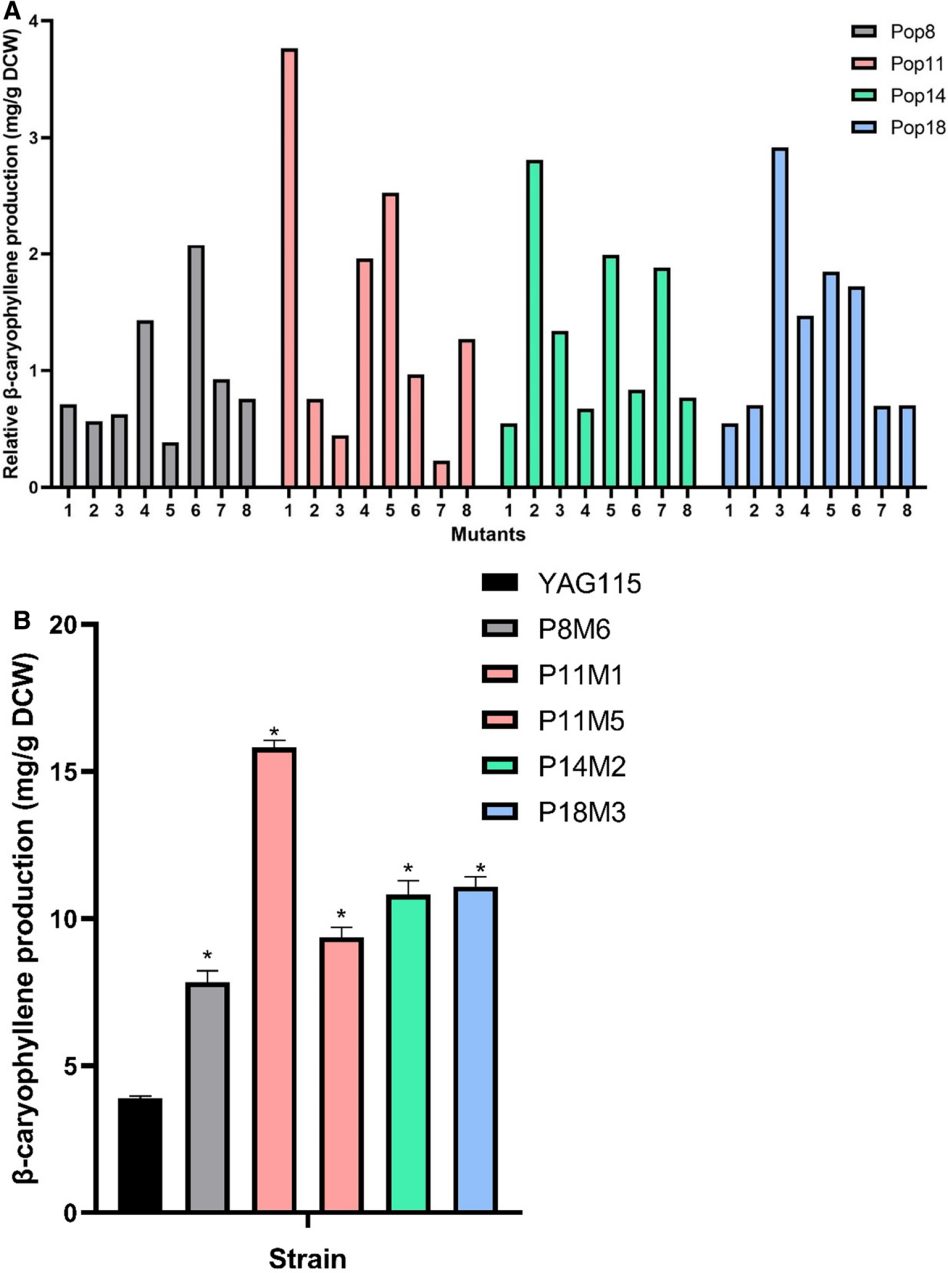
Isolated evolved mutants exhibited increased β-caryophyllene production over the parental strain. **A** Initial screening of isolated mutants from each evolved population based on relative product yield in 48 well plates. Pop8 = P2 (day 8), Pop11 = P2 (day 11), Pop14 = P2 (day 14), and Pop18 = P2 (day 18). Production was measured in 48 well plate with 1 mL culture overlayed with 166 μL dodecane. **B** Confirmation of product formation in the five best performing mutants. Production was measured in 20 mL test tube with 3 mL culture overlayed with 500 μL dodecane. The mutants are named first by the population they were isolated from followed by the isolate number. For example, P11M1 is the first mutant from Pop11. Asterisks: p value < 0.05 using two-tailed Student’s t-test against control

Using a 30-min exposure to 1 M hydrogen peroxide, we subjected four time point samples from the evolved population: P1 (day 4), P2 (day 8), P2 (day 11), P2 (day 14) and P2 (day 18) to screen for hyperproducers. We failed to identify any viable isolates from population P1 (day 4). For the remaining populations, eight individual colonies per population were randomly chosen. The isolated mutants were subjected to a second round of screening based on their β-caryophyllene production. Figure 3A shows the individual production obtained from each isolate. The top five overproducing evolved mutants were selected from the 32 total mutants, and their β-caryophyllene production were characterized in more detail. Results showed a two to fourfold increase in production compared to the parental strain, with mutant P11M1 (population 2 day 11 mutant 1) being the best performer as shown in Fig. 3B. The stability of P11M1 was assessed via four serial passages in SC-ura-leu media (3 days of growth each passage for a total of 12 days); β-caryophyllene production was monitored after each passage. The parental strain was included as control. P11M1 showed consistent production of ~ 16 mg/g DCW, whereas the control produced ~ 4 mg/g DCW.

**Fig. 4:**
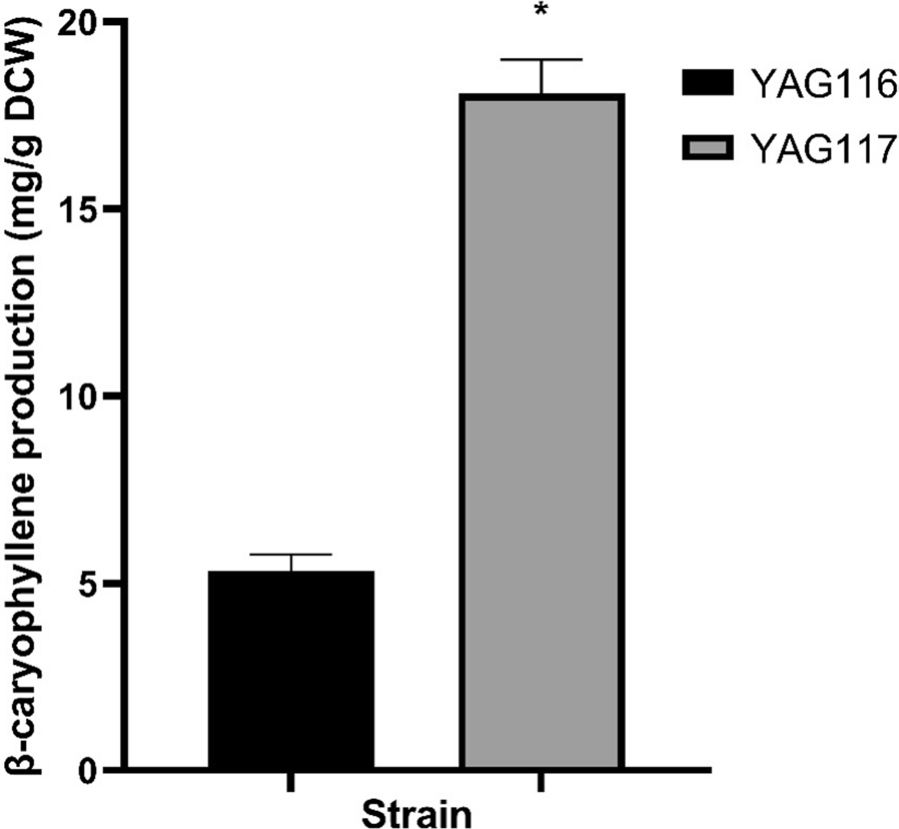
Production of β-caryophyllene in strains with additional copies of *QHS1* gene. Production was measured in 20 mL test tube with 3 mL culture overlayed with 500 μL dodecane. Asterisks: p value < 0.05 using two-tailed Student’s t-test compared with YAG116

To determine whether *QHS1* is the rate-limiting step in β-caryophyllene production in the evolved mutants, the *QHS1* gene was overexpressed using 2μ plasmid into both the parental and the best evolved isolate P11M1, resulting in strain YAG116 and YAG117, respectively. Overexpression of *QHS1* led to modest increases in production in the parental strain from ~ 4 mg/g DCW (20.1 ± 0.3 mg/L) to ~ 5.3 mg/g DCW (27.8 ± 2.1 mg/L) and P11M1 from ~ 16 (89.7 ± 2.4 mg/L) to ~ 18 mg/g DCW (104.7 ± 6.2 mg/L) (Fig. 4), suggesting that β-caryophyllene synthase is not a major rate-limiting step in β-caryophyllene production in the evolved mutants.

### Genome sequencing identified two beneficial mutations for β-caryophyllene production

**Fig. 5:**
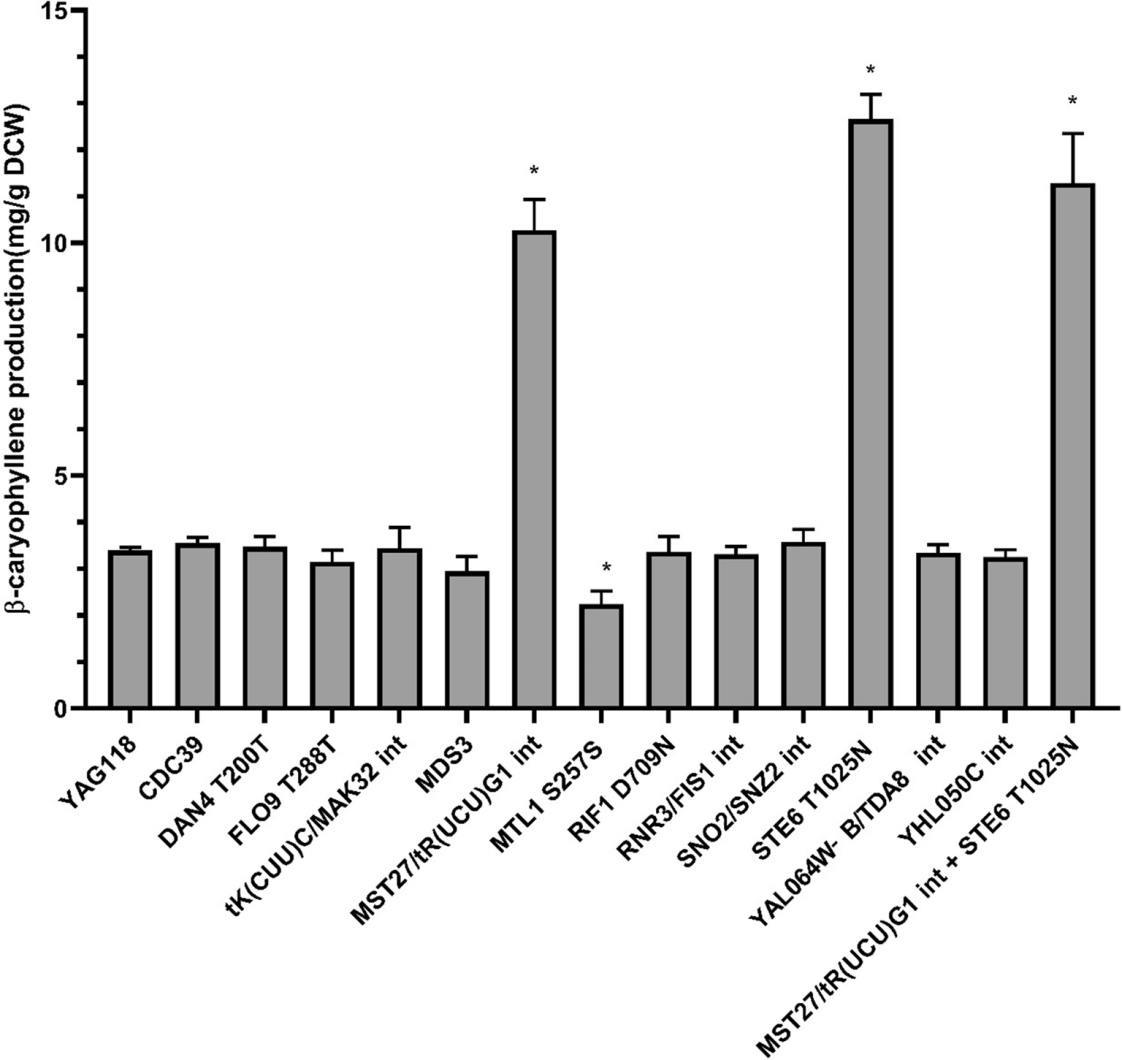
Production of β-caryophyllene in reconstructed single mutants. Production was measured in 20 mL test tube with 3 mL culture overlayed with 500 μL dodecane. Asterisks: p-value < 0.05 using two-tailed Student’s t-test against control

To identify beneficial mutations for the observed increased production in the isolated mutants, we sequenced four time-course population samples from population P2 (day 8, day 11, day 14 and day 18), five of best isolated mutants, and the parental strain. A mutation frequency cutoff of greater than or equal to 0.8 was used to narrow down the dominant mutations in the population. Large numbers (> 200) of mutations above the cutoff threshold were observed in the population samples. The identified mutations in single hyperproducer isolates are shown in Additional file 1: Table S3. The high frequency mutations (in populations) that were also identified in sequenced single isolates from the respective populations were selected for further analyses. In addition, since isolate P11M1 was the best producer identified, all mutations in P11M1 were also chosen, for a total of 13 unique mutations selected for further analyses (Table 1). To determine the impacts of each of these mutations on β-caryophyllene biosynthesis, single mutations were introduced into parental strain using site-directed mutagenesis, resulting in strains YAG132-145. Only two mutations led to improved production, the mutation in *STE6* gene and a mutation in intergenic region of between *MST27/tR(UCU)G1* (Fig. 5 and Additional file 1: Table S4). *STE6 T1025N* mutation improved production 3.7 fold to 12.6 mg/g DCW of β-caryophyllene. The *MST27/tR(UCU)G1* intergenic mutation improved production threefold to 10.3 mg/g DCW of β-caryophyllene compared to the parental strain. No further increase in production was observed when these two mutations were combined.

**Table 1:** Mutations chosen for detailed characterization

Chromosome	Position	Mutation	Amino acid change	Gene	Abbreviation
3	286312	C→T	Nonsense mutation	CDC39	CDC39
10	715141	A→G	T200T	DAN4	DAN4 T200T
1	27105	A→G	T288T	FLO9	FLO9 T288T
3	151555	+ A	Intergenic	tK(CUU)C/MAK32	tK(CUU)C/MAK32 int
7	128474	T→A	Nonsense mutation	MDS3	MDS3
7	404475	+ G	Intergenic	MST27/tR(UCU)G1	MST27/tR(UCU)G1 int
7	530034	A→C	S257S	MTL1	MTL1 S257S
2	754982	C→T	D709N	RIF1	RIF1 D709N
9	241053	(A)_21→22_	Intergenic	RNR3/FIS1	RNR3/FIS1 int
14	12986	(T)_11→14_	Intergenic	SNO2/SNZ2	SNO2/SNZ2 int
11	43222	G→T	T1025N	STE6	STE6 T1025N
1	12690	A→T	Intergenic	YAL064W-B/TDA8	YAL064W-B/TDA8 int
8	2303	(C)_11→12_	Intergenic	YHL050C/YHL050C	YHL050C int

All mutations were verified by Sanger sequencing

### Growth kinetics and oxidative tolerance of reconstructed mutations

In order to investigate the general fitness benefit conferred by the individual mutations identified, we did a growth kinetics study for all individual reconstructed strains. The data showed that five (*CDC39*, *tK(CUU)C/MAK32 int, RIF1 D709N, STE6 T1025N* and *YAL064W B/TDA8 int*) out of 13 mutations did not impact growth under normal culture conditions (see Table 2)*.* The *FLO9 T288T, RNR3/FIS1 int*, and *SNO2/SNZ2 int* mutations exhibited increased growth rates. The *DAN4 T200T, MDS3, MTL1 S257S, MST27/tR(UCU)G1 int*, and* FLO9 T288T* mutations showed significant reduction in lag phase.

**Table 2:** Growth kinetics data for reconstructed strains

Strain	Mutations	Growth rate (h^−1^)	Stdev	Lag phase (h)	Stdev	OD600Max	Stdev
YAG118	Control	0.17	0.01	3.35	0.14	1.05	0.02
YAG132	CDC39	0.18	0.01	3.34	0.27	1.09	0.06
YAG133	DAN4 T200T	0.18	0.02	**2.71**	**0.28**	1.04	0.05
YAG134	FLO9 T288T	**0.20**	**0.01**	**2.19**	**0.38**	**1.15**	**0.01**
YAG135	tK(CUU)C/MAK32 int	0.16	0.02	3.43	0.15	0.99	0.09
YAG136	MDS3	0.18	0.01	**0.72**	**0.14**	1.07	0.04
YAG137	MST27/tR(UCU)G1 int	0.17	0.01	**2.39**	**0.10**	1.06	0.04
YAG138	MTL1 S257S	0.18	0.01	**1.12**	**0.15**	1.05	0.05
YAG139	RIF1 D709N	0.19	0.01	3.37	0.11	1.09	0.02
YAG140	RNR3/FIS1 int	**0.20**	**0.01**	3.20	0.10	1.12	0.06
YAG141	SNO2/SNZ2 int	**0.20**	**0.01**	3.21	0.02	**1.18**	**0.02**
YAG142	STE6 T1025N	0.18	0.01	3.15	0.23	1.08	0.08
YAG143	YAL064W-B/TDA8 int	0.17	0.03	3.18	0.35	1.10	0.14
YAG144	YHL050C int	0.17	0.01	**3.68**	**0.03**	1.07	0.06
P11M1	Multiple	0.19	0.01	**2.34**	**0.18**	1.11	0.05

Bold: p value < 0.05 using two-tailed Student’s t-test compared with control

To assess the impacts of these mutations on oxidative stress tolerance, the individual reconstructed mutants (β-caryophyllene producers) were subjected to 200 mM hydrogen peroxide exposure for 30 min. The same set of mutations (listed in Table 1) were also reconstructed in a background strain lacking the β-caryophyllene synthase gene, resulting in strains YAG119–YAG131, which were used to study the impacts of these mutations on oxidative stress tolerance in the absence of production. In the absence of β-caryophyllene production, an increased 10X survival compared to the reference strain (YAG114) was observed in YAG121 (*FLO9 T288T*), YAG123 (MDS3), YAG124 (*MST27/tR(UCU)G1 int*) and YAG125 (*MTL1 S257S*) (Table 3). In the β-caryophyllene producing background, 10X increased tolerance was observed in YAG134 (*FLO9 T288T*), YAG136 (MDS3), YAG137 (*MST27/tR(UCU)G1 int*), YAG138 (*MTL1 S257S*) and YAG142 (*STE6 T1025N*).

**Table 3:** Hydrogen peroxide stress tolerance for strains with and without β-caryophyllene production after 200 mM H_2_O_2_ exposure for 30 min

Mutation	No production		With production		
	Strain	Relative H_2_O_2_ tolerance	Strain	Relative H_2_O_2_ tolerance	β-caryophyllene (mg/g DCW)
Control	YAG114		YAG118		3.39
CDC39	YAG119	ND	YAG132	ND	3.55
DAN4 T200T	YAG120	ND	YAG133	ND	3.47
FLO9 T288T	YAG121	+	YAG134	+	3.15
tK(CUU)C/MAK32 int	YAG122	ND	YAG135	ND	3.44
MDS3	YAG123	+	YAG136	+	2.94
MST27/tR(UCU)G1 int	YAG124	+	YAG137	+	**10.27**
MTL1 S257S	YAG125	+	YAG138	+	2.24
RIF1 D709N	YAG126	ND	YAG139	ND	3.36
RNR3/FIS1 int	YAG127	ND	YAG140	ND	3.31
SNO2/SNZ2 int	YAG128	ND	YAG141	ND	3.57
STE6 T1025N	YAG129	ND	YAG142	+	**12.66**
YAL064W-B/TDA8 int	YAG130	ND	YAG143	ND	3.35
YHL050C int	YAG131	ND	YAG144	ND	3.25

Production was measured in 20 mL test tube with 3 mL culture overlayed with 500 μL dodecane. ND: not different from wild-type. +: ~ 10× higher survival compared to wild-type. Bold: p value < 0.05 using two-tailed Student’s t test compared with control

### Effect of *STE6 T1025N* on caryophyllene production

**Fig. 6:**
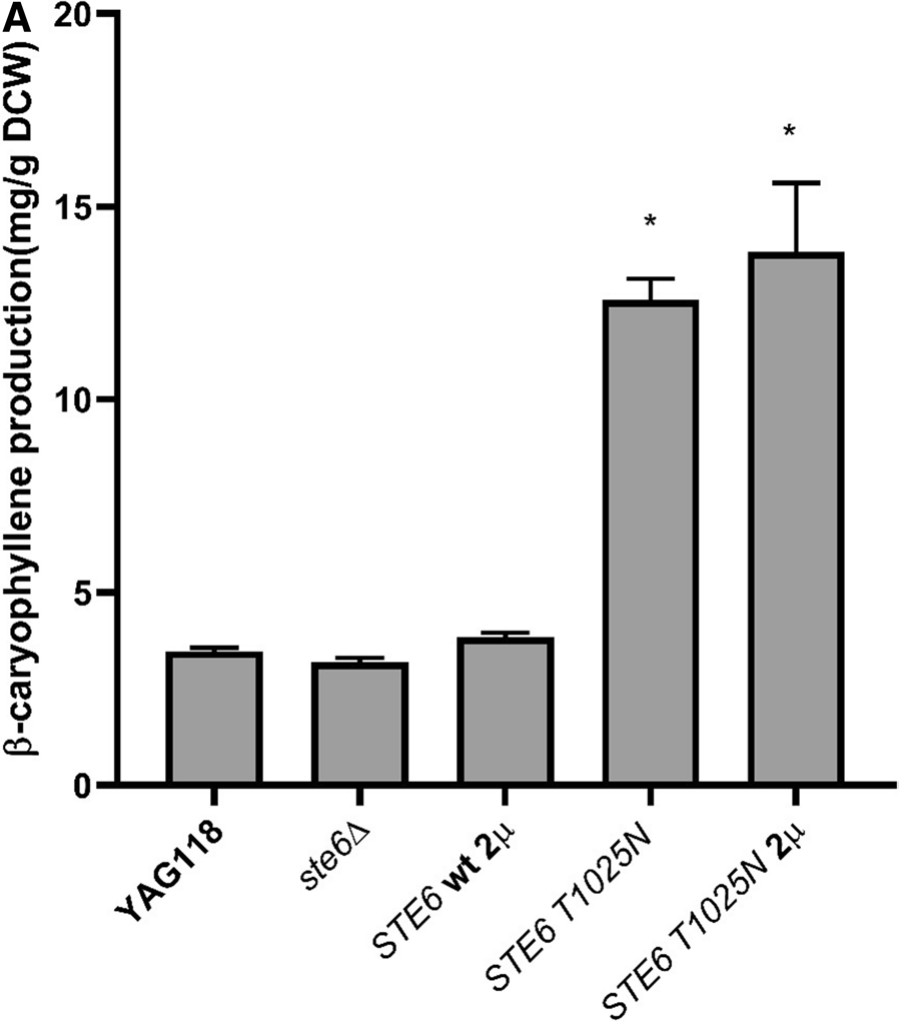
The effect of *STE6* expression on β-caryophyllene production. *ste6*∆: *ste6* deletion strain. *STE6* wt 2µ: wild-type *STE6* expressed on a 2µ plasmid. *STE6 T1025N*: reconstructed parental strain with *STE6 T1025N* mutation. *STE6 T1025N* 2µ: parental strain expressing mutated *STE6* gene on a 2µ plasmid. Production was measured in 20 mL test tube with 3 mL culture overlayed with 500 μL dodecane. Asterisk: p value < 0.05 using two-tailed Student’s t-test against parental strain

Since the missense mutation in *STE6* led to a large impact on β-caryophyllene production, we assessed whether the mutation is a loss-of-function or gain-of-function mutation using *STE6* knockout (YAG146) and overexpression (YAG147 with *STE6 T1025N* and YAG148 with wild-type *STE6*) strains, and quantifying their effects on β-caryophyllene production (data shown in Fig. 6). The *STE6* knockout strain exhibited no change in production compared with the reference. Production was also not impacted when the wild type *STE6* was overexpressed. However, when we overexpressed the mutated *STE6* in the reference strain, β-caryophyllene production increased fourfold to 13.8 mg/g DCW, suggesting the *STE6 T1025N* mutation is a gain-of-function mutation.

We further explored the possibility that the *STE6 T1025N* mutation may influence the efflux and thus the production of other sesquiterpenes by overexpressing the mutated gene in an engineered α-humulene producer. No increase in production was observed in the strain that overexpresses *STE6 T1025N* compared to the reference (Additional file 1: Figure S6), suggesting that the mutation specifically affects the transport of β-caryophyllene and does not significantly influence the efflux of sesquiterpenes in general.

## Discussion

β-Caryophyllene was produced in yeast using heterologous *QHS1* gene and production was further improved using overexpression of intermediate FPP. Periodic (30 min) and continuous exposure to hydrogen peroxide at various concentrations demonstrated the benefit of β-caryophyllene production on oxidative stress protection. Leveraging this oxidative stress protection of β-caryophyllene, two schemes of selective pressure were evaluated for 8 days for improving production. Continuous exposure resulted in no significant increase in production whereas periodic exposure resulted in 1.7-fold increment in production. Periodic challenge strategy was used for ALE experiment and resulted in threefold increment in production. Isolated mutants showed a correlation between size and production suggesting a growth advantage of mutants with higher production of β-caryophyllene.

Thirteen unique mutations were found through next-gen sequencing in population samples and isolated mutants. *STE6 T1025N* and intergenic mutation between *MST27/tR(UCU)G1* showed the highest production improvement. *STE6* encodes the plasma membrane ATP binding cassette (ABC) transporter known to export a-factor in MATa cells [22], and is a homolog to the mammalian multidrug resistance *mdr* gene [23]. Bacteria commonly acquire mutations in efflux pumps to overcome stress conditions while producing terpenoids [24, 25]. The *STE6 T1025N* mutation is a missense mutation, resulting in a change in amino acid from threonine to asparagine. A mutation in *STE6 K1093A*, which lies within a nucleotide binding domain, has previously been reported to increase the a-factor export in *STE6* [26]. The *STE T1025N* mutation identified here is not located in any of the predicted nucleotide binding domains, and is 62 amino acids upstream of a conserved Walker A motif [27, 28]*.* Since the mutation is located between the transmembrane and ATP binding domains on the predicted cytoplasmic portion of the protein, it potentially impacted substrate recognition, allowing increased export of β-caryophyllene. However, further experiments are needed to identify the exact effect the mutation has on transport of β-caryophyllene. The *MST27/tR(UCU)G1 int* mutation is an insertion mutation between *MST27* and a Ty element [29]. *MST27* is member of the *DUP240* multi-gene family and known to impact the vesicles formation [30]. Ty1 elements is a transposon element and mutations in transposon elements are commonly found in evolution experiments and are known to impact fitness [31, 32]. However, as this mutation is in the intergenic region and at the 3ʹ end of *MST27*, it is difficult to ascertain the exact impact of the mutation on the cell.

Interestingly, both the *STE6 T1025N* and *MST27/tR(UCU)G1* intergenic mutations were found in very low frequencies in the population (below detection limit). The *STE6 T1025N* mutation was found only in mutant P11M1 and *MST27/tR(UCU)G1* intergenic mutation was identified in all the isolated mutants sequenced. Note that the population samples sequenced were cultured in normal growth conditions before harvesting for next-gen sequencing, while the isolated mutants underwent a round of selection in 1 M hydrogen peroxide. The findings that the *MST27/tR(UCU)G1* intergenic mutation was not detected in the population samples, but is present in all isolated mutants, and the positive correlation between cellular survival (based on colony size after H_2_O_2_ challenge; Additional file 1: Figure S5) and product formation, suggest that the evolving population was highly heterogeneous with the most productive members consisting of a smaller fraction of the population.

Mutation in *FLO9 T288T* positively impacted specific growth rate, lag phase, and max OD600, suggesting a significant fitness benefit, which likely allowed it to reach fixation in the evolving population. The synonymous mutation in *FLO9* changed the codon for threonine from ACT to the less preferred ACC, which may impact translation of the protein. Mutations and changes in copy number in the *FLO* genes have been found to be involved in tolerance to environmental stressors [33–35]. Mutations in all *FLO9* and *MDS3* were found in detectable frequencies in all populations sequenced, and the *MTL1* mutation was present at ~ 49% frequency in the P11 population, suggesting that the increased oxidative stress tolerance conferred by these mutations likely contributed to their selection in the ALE experiment. The *MTL1 S257S* mutation is also a synonymous mutation, with a serine codon change from TCA to a less preferred TCC, suggesting this mutation may lead to reduced translation of the protein. Mutations in *FLO9* and *MTL1* were found in an industrial yeast strain that was evolved for growth on hydrolysates inhibitors [33], which have been shown to induce oxidative stress in *S. cerevisiae* [36]. The increased hydrogen peroxide tolerance in YAG142 (*STE6 T1025N*) can be attributed to increased production, as without β-caryophyllene production there was no benefit to survival in oxidative stress challenge. The intergenic mutation in *MST27/tR(UCU)G1*, which is one of the two mutations found to be responsible for increased β-caryophyllene production, showed general fitness benefit with reduced lag phase in normal growth conditions and ~ 10× increase in survival in the presence of hydrogen peroxide stress. While this mutation is likely selected for due to the fitness it confers in the presence of strong oxidative stress, although it’s frequency in the population is extremely low, it is unclear how it contributed to increased β-caryophyllene production.

## Conclusions

In this work, we explored the use of adaptive laboratory evolution to improve the production of an extracellular product, β-caryophyllene, using oxidative stress challenge as selection. Initial metabolic engineering by overexpressing isoprenoid pathway genes resulted in a parental strain that produces 3–4 mg/g DCW of β-caryophyllene. An ALE strategy was optimized by comparing periodic stress challenge versus continuous exposure. Using the optimized periodic H_2_O_2_ challenge method (from 50 to 200 mM H_2_O_2_), the ALE experiment resulted in evolving populations with up to 15.8 mg/g DCW (> threefold increase) in β-caryophyllene production. Under the assumption that a positive correlation exists between oxidative stress tolerance and β-caryophyllene production, a final selection using 1 M H_2_O_2_ for 30 min was used to isolate mutants with the highest level of oxidative stress tolerance. This strategy yielded isolated mutants with up to fourfold increase in β-caryophyllene production. Mutations in the intergenic region of *MST27/tR(UCU)G1* and a non-synonymous mutation in *STE6* were the only mutations found to benefit product formation. *STE6* is a known ABC exporter for a-factor in yeast. Deletion and overexpression studies demonstrated that the mutation is a gain-of-function mutation. It may be possible that the *STE6 T1025N* mutation resulted in binding and export of β-caryophyllene. However, the exact mechanism for how this point mutation is affecting β-caryophyllene production is still unclear. Overall, we demonstrated that by leveraging the antioxidant potential of terpenes that are exported extracellularly, product formation can be coupled with cellular growth/survival and be rapidly improved using short-term ALE experiments.

## Materials and methods

### Strains, plasmids and growth conditions

All yeast strains used in this work are derivatives of S288c and listed in Additional file 1: Table S5. *S. cerevisiae* strain BY4741 (MATa, his3Δ1, leu2Δ0, met15Δ0, ura3Δ0) [37] are used as the base strains in this study. Yeast strains are cultured in Synthetic Complete (SC) media lacking specific amino acids for selection at 30 °C. *E. coli* strains used for subcloning were cultured at 37 °C in Luria Broth (LB) supplemented with appropriate antibiotics. Cytosolic catalase T (*CTT1*) gene was deleted in BY4741 strain as described in our earlier work [18]. For terpene quantification, yeast strains were cultured in YPD at 30 °C and 200 rpm for 72 h.

### Plasmid construction for QHS1 and FPP overproduction

The heterologous genes *QHS1* from *Artemisia annua* [11] were codon optimized for *S. cerevisiae* and synthesized (Integrated DNA Technologies). Codon optimized *QHS1* gene was integrated into the yeast genome or expressed on plasmid under *URA3* selection marker. To increase flux towards FPP, *tHMG1, HMG2(K6R), UPC2-1* and *ERG20* were PCR amplified from yeast genome and added to chromosome at *LEU2* locus under *LEU2* selection. The truncated *HMG1*, *tHMG1*, contains the1575-bp C-terminal part of *HMG1* and was amplified from yeast genome using PCR and a start codon was added in the forward primer. The plasmids were constructed using the MoClo-YTK plasmid kit [38] (Addgene). In brief, each gene was introduced into the entry vector YTK001 using BsmBI (Thermo Scientific) via Golden Gate assembly protocol described by [38]. Each gene (in an entry vector) is then assembled into transcriptional unit plasmid along with the appropriate promoter and terminator part vectors using BsaI (Thermo Scientific) via Golden Gate assembly. Finally, different transcriptional units are joined together to be used as plasmid or genomic integration with different selection markers for yeast. DH5α chemically competent *E. coli* cells were used for cloning and were prepared using Zymo Mix & Go! E. coli Transformation Kit and Buffer Set™ (Zymo Research). Yeast competent cells were prepared using Frozen-EZ Yeast Toolkit II Kit ™ (Zymo Research). To produce α-humulene *ZSS1* from *Zingiber zerumbet* was codon optimized for *S. cerevisiae* and synthesized (Integrated DNA Technologies) and introduced into genome as described above. pTDH3 was used as promoter and tTDH1 was used as terminator for *ZSS1.*

### Quantification of β-caryophyllene production

Organic layer of dodecane on top of media was used to capture volatile β-caryophyllene produced by strains [39]. Unless specified a ratio of six-part media and one-part dodecane was used. A volume of 100 μL of the dodecane overlay was sampled after 3 days or 24 h (for evolution experiment) for use in product quantification. Quantitative analysis of β-caryophyllene in dodecane layer was performed using gas chromatography [40]. Alpha-humulene was used as internal standard for peak normalization. An Agilent J&W HP-5 (5%-phenyl)-methylpolysiloxane nonpolar column (30 m × 0.32 mm with 0.25 μm film thickness) was used for this study. Gas chromatogram oven temperature was programmed from 100 °C initial temperature to 140 °C at 10 °C/min rate, followed by 2.5 °C/min to 180 °C, followed by 20 °C/min until final temperature of 200 °C. FID detector was kept at 280 °C whereas inlet was kept at 240 °C in a split-less mode. Flow was kept at 2 mL/min with hydrogen flow at 30 mL/min and ultra-pure air at 400 mL/min. Nitrogen was used for makeup flow at 25 mL/min. At least three biological replicates per strain were used for analysis.

### Growth kinetics measurements

Microplate reader (TECAN Infinite ® M Nano) was used to measure growth curves for strains. Cells were grown for 24 h in test tubes, then normalized to OD_600_ ~ 0.05 in 200 μL final volume in media in 96-well plates. Cells were cultured in the microplate reader for 72 h with orbital shaking at constant intervals using kinetic cycles of 2 min incubation time, then orbital shaking for 3 min at 198 rpm (3 mm amplitude) followed by OD600 measurements. To obtain specific growth rate µ, duration of growth lag, and the maximum OD600, software grofit v1.1.1 [41] was used. Input file for grofit was generated and following script was ran in R:

growthdata <- read.csv("inputfile.csv",sep=",", header=TRUE, check.names = FALSE)

timedata <- read.csv("timeworksheet.csv", sep=",", header=TRUE, check.names = FALSE)

gro <- grofit(timedata, growthdata)

summary_table <- summary.gcFit(gro$gcFit)

write.csv(summary_table,"Summary.csv")

When prompted to choose the models out of four preexisting model (logistic, richards, gompertz, or gompertz.exp), the models which fit best the growth curve was selected. Results were compiled from the summary table obtained from the software. Three biological replicates per strain were used for analysis.

### Oxidative stress tolerance using hydrogen peroxide

For spot assays, overnight cultures were normalized to OD 600 of 1.0 using phosphate-buffered saline (PBS). Strains were treated with hydrogen peroxide for 30 min intervals in a shaking incubator at 30 °C at 200 rpm. Cells were washed two times with PBS to eliminate the remaining hydrogen peroxide in solution. After the shock treatment, tenfold serial dilutions were performed in (PBS) and spotted on SC plates lacking appropriate amino acid. Three biological replicates per strain were used for analysis. For stress optimization and evolution experiment, the normalization step was omitted from the protocol.

### Adaptive laboratory evolution

Single colonies of YAG115 were used to initiate the evolution experiment in 3 mL SC-ura-leu media on day 0. For populations that were evolved using the constant exposure strategy, 200 µL of cells were inoculated in fresh media supplemented with specified concentrations of H_2_O_2_. For populations that were subjected to periodic challenge, on day 1, 500 μL of culture was centrifuged, resuspended in phosphate buffer saline (PBS) and subjected to 50 mM H_2_O_2_ challenge for 30 min under shaking (challenge period) and washed twice with PBS after exposure. Population samples were preserved in glycerol stocks each day. 200 μL of challenged cells were inoculated in 3 mL of SC-ura-leu media and 500 μL of dodecane was added for β-caryophyllene capture. On day 2, the population was allowed to recover by transferring 200 μL of overnight culture into fresh media (recovery period). The populations were challenged on odd days with specified concentration of H_2_O_2_ and allowed to recover on even days. Production was quantified at the end of 24-h by recovering the dodecane layer for populations evolved using either the continuous or periodic challenge strategies.

### Isolating mutants from evolved populations

To select for hyperproducing mutants from the evolved populations, each population was subjected to exposure to a higher concentration of hydrogen peroxide than that used during the ALE experiments. 500 μL of each population sample was centrifuged and cells were resuspended in PBS with final concentration of 1 M of hydrogen peroxide. After 30 min of exposure, the cells were washed 2 time with PBS, and all cells were plated on SC-ura-leu plates. Plates were kept for 2 days at 30 °C. 8 colonies were randomly picked from each challenged population and streaked on SC-ura-leu plates to ensure we obtain individual clones. Single colonies were picked and cultured in 48 well plates containing 1 mL of media and ~ 166 μL dodecane overlay at 30 °C and shaking at 200 rpm. The dodecane layer was recovered to quantify β-caryophyllene production after 72 h. To confirm the 48 well culture results, the high performing clones were grown in 3 mL cultures in test tubes with 500 μL dodecane layer using three biological replicates.

### Whole genome resequencing

Genomic DNA of isolated mutants, parental strain and population samples were extracted using YeaStar DNA extraction kit (Zymo Research). Library preparations and NGS sequencing were performed by the Texas A&M Genomics Center for sequencing on the Illumina MiSeq platform using 300 × 300 paired-end reads using Nexterra DNAFlex kit for library generation. An average coverage of > 20-fold was obtained for each isolated mutant and > 150-fold for population samples. The sequencing data was aligned to *S. cerevisiae* S288c reference genome with breseq v0.29 [42]. De novo mutations in isolated mutants were identified by comparing against the YAG113 parental strain, and verified via Sanger sequencing. The raw sequencing data were deposited in SRA database (https://www.ncbi.nlm.nih.gov/sra) with accession number PRJNA669136.

### Reconstructing mutations into YAG114 and YAG118 strain

CRISPR–Cas9 using the one plasmid-system with pCRCT developed by [43] was used for site-directed mutagenesis to reconstruct identified mutations into YAG118. Briefly, 120 bp sequence including donor sequence and guide sequence (Additional file 1: Table S6) was chemically synthesized by Twist Bioscience, USA, then introduced into the pCRCT plasmid using Golden Gate reaction using BsaI restriction sites [44], and transformed into *E. coli* cells, and plated on LB + Xgal for blue/white screening and incubated overnight at 37 °C. The bacterial colony with correct plasmid construction was verified by restriction digestion. The constructed CRISPR–Cas9 plasmid was then transformed into YAG114 and YAG118 strains and selected on SC-uracil plates. Colonies were picked and target mutations were first verified using PCR amplification refractory mutation system (ARMS) as described by [45] followed by confirmation via Sanger sequencing. Cas9 plasmid was cured by serially passaging the strain in YPD 3 times and verified by PCR amplification.

## Supplementary Information

**Additional file 1.** List of Additional tables and figures.

## Data Availability

The datasets during and/or analysed during the current study available from the corresponding author on reasonable request.

## Abbreviations

ALE: Adaptive laboratory evolution
FPP: Farnesyl pyrophosphate
PCR: Polymerized chain reaction
SC: Synthetic complete
DCW: Dry cell weight
