# Supplementary material for: Adaptive laboratory evolution of β-caryophyllene producing Saccharomyces cerevisiae
Source: Microb Cell Fact. 2021 May 27;20:106. doi: 10.1186/s12934-021-01598-z (PMC8157465; doi:10.1186/s12934-021-01598-z)
Supplement: Supplementary file 1 — Additional file 1. List of Additional tables and figures. [file 12934_2021_1598_MOESM1_ESM.docx]

**List of Additional Tables**

Table S1: Table S1: Production of strain with just QHS1 gene (YAG111) and strain with QHS1 and FPP overproduction genes (YAG115) integrated into genome. Production was measured in 20 ml test tube with 3 ml culture overlayed with 500 μl dodecane.

| Strain | β-caryophyllene (mg/g DCW) | Standard deviation |
| --- | --- | --- |
| YAG111 | 0.79 | 0.09 |
| YAG115 | 3.09 | 0.52 |

Table S2: Growth kinetics for strains with continuous exposure to hydrogen peroxide. Cells were grown in 96 well plate in a microplate reader for 72 hr and growth curves were calculated using grofit v1.1.1. NG: No Growth observed

| **Strain** |  | **Control** | **25mM** | **50mM** | **75mM** | **100mM** |
| --- | --- | --- | --- | --- | --- | --- |
| **BY4741** | **μ (h^-1^)** | 0.09 ± 0.00 | 0.04 ± 0.00 | 0.05 ± 0.01 | 0.02 ± 0.01 | NG |
|  | **Lag phase (h)** | 4.80 ± 0.20 | 27.24 ± 0.12 | 31.30 ± 0.09 | 27.58 ± 0.31 | NG |
|  | **Max OD** | 1.10 ± 0.01 | 0.72 ± 0.00 | 0.85 ± 0.00 | 0.48 ± 0.01 | NG |
| **YAG110** | **μ (h^-1^)** | 0.10 ± 0.01 | NG | NG | NG | NG |
|  | **Lag phase (h)** | 4.61 ± 1.14 | NG | NG | NG | NG |
|  | **Max OD** | 1.13 ± 0.10 | NG | NG | NG | NG |
| **YAG114** | **μ (h^-1^)** | 0.05 ± 0.00 | 0.02 ± 0.00 | 0.01 ± 0.00 | NG | NG |
|  | **Lag phase (h)** | 3.63 ± 0.32 | 12.35 ± 2.16 | 26.50 ± 3.32 | NG | NG |
|  | **Max OD** | 0.47 ± 0.02 | 0.33 ± 0.03 | 0.15 ± 0.01 | NG | NG |
| **YAG111** | **μ (h^-1^)** | 0.05 ± 0.00 | 0.04 ± 0.00 | 0.02 ± 0.00 | 0.02 ± 0.00 | NG |
|  | **Lag phase (h)** | 4.19 ± 0.11 | 14.82 ± 0.32 | 18.60 ± 1.28 | 33.68 ± 2.40 | NG |
|  | **Max OD** | 0.70 ± 0.03 | 0.52 ± 0.04 | 0.30 ± 0.03 | 0.22 ± 0.04 | NG |
| **YAG115** | **μ (h^-1^)** | 0.04 ± 0.00 | 0.03 ± 0.00 | 0.02 ± 0.00 | 0.01 ± 0.00 | 0.01 ± 0.00 |
|  | **Lag phase (h)** | 3.89 ± 0.15 | 11.46 ± 0.25 | 12.35 ± 0.54 | 12.81 ± 2.00 | 15.09 ± 1.51 |
|  | **Max OD** | 0.48 ± 0.02 | 0.40 ± 0.02 | 0.29 ± 0.01 | 0.24 ± 0.02 | 0.20 ± 0.00 |

Table S3: List of mutations found, frequency of each mutation in corresponding population sample is also shown. Each of these mutations were further verified using Sanger sequencing. Not available: mutation was not confirmed in Sanger sequencing. Dash - not considered for sequencing

| Mutant | Frequency in population | Chromosome | Position | Mutation | Annotation | Gene | Sanger confirmation |
| --- | --- | --- | --- | --- | --- | --- | --- |
| P8M6 | 0 | 14 | 12986 | (T)_11→14_ | intergenic (‑110/‑281) | SNO2 ← / → SNZ2 | - |
|  | 0.94 | 1 | 26973 | A→G | F332F (TTT→TTC) | FLO9 ← | Confirmed |
|  | 0.95 | 1 | 26981 | T→C | S330G (AGC→GGC) | FLO9 ← | Confirmed |
|  | 0.94 | 1 | 26983 | T→G | N329T (AAC→ACC) | FLO9 ← | Confirmed |
|  | 0.71 | 1 | 27090 | T→C | E293E (GAA→GAG) | FLO9 ← | - |
|  | 0.79 | 1 | 27105 | A→G | T288T (ACT→ACC) | FLO9 ← | - |
|  | 0 | 13 | 87053 | (TAT)_36→34_ | intergenic (‑314/+65) | PRE8 ← / ← RPM2 | - |
|  | 0.13 | 7 | 128474 | T→A | Y1259* (TAT→TAA) | MDS3 → | - |
|  | 0 | 9 | 265719 | G→C | T702S (ACT→AGT) | SYG1 ← | - |
|  | 0 | 4 | 273653 | (T)_24→23_ | coding (1224/1224 nt) | QRI7 ← | - |
|  | 0 | 7 | 400571 | (T)_21→22_ | intergenic (+411/+300) | OLE1 → / ← ERV14 | - |
|  | 0 | 4 | 403520 | (T)_9→10_ | intergenic (‑229/+175) | MPS1 ← / ← MRX9 | - |
|  | 0 | 7 | 404475 | +G | intergenic (+84/‑995) | MST27 → / → tR(UCU)G1 | - |
|  | 0 | 12 | 468701 | T→C | intergenic (‑1832/‑112) | RDN18‑2 ← / → RDN5‑2 | - |
|  | 0 | 7 | 531875 | (TA)_15→17_ | intergenic (+194/+8) | tD(GUC)G1 → / ← THG1 | - |
|  | 0 | 4 | 548565 | (T)_19→20_ | intergenic (‑255/+197) | RPC11 ← / ← BAP3 | - |
|  | 0.64 | 13 | 908174 | 2 bp→TC | coding (811‑812/3423 nt) | YMR317W → | - |
|  | 0.65 | 13 | 908177 | A→G | I272V (ATT→GTG) | YMR317W → | - |
|  | 0.67 | 13 | 908179 | T→G | I272V (ATT→GTG) | YMR317W → | - |
|  | 0.63 | 13 | 908185 | A→G | S274S (TCA→TCG) | YMR317W → | - |
|  | 0.68 | 13 | 908196 | G→C | W278S (TGG→TCG) | YMR317W → | - |
|  | 0.65 | 13 | 908198 | G→T | A279S (GCA→TCA) | YMR317W → | - |
|  | 0.61 | 13 | 908203 | G→A | T280T (ACG→ACA) | YMR317W → | - |
|  | 0.66 | 13 | 908218 | C→T | S285S (AGC→AGT) | YMR317W → | - |
| P11M1 | 0 | 8 | 2303 | (C)_11→12_ | intergenic (‑406/+368) | YHL050C ← / ← YHL050C | Confimed |
|  | 0.36 | 1 | 12690 | A→T | intergenic (+264/+673) | YAL064W‑B → / ← TDA8 | Confirmed |
|  | 0 | 14 | 12986 | (T)_11→14_ | intergenic (‑110/‑281) | SNO2 ← / → SNZ2 | Confimed |
|  | 0.83 | 1 | 27105 | A→G | T288T (ACT→ACC) | FLO9 ← | Confirmed |
|  | 0 | 11 | 43222 | G→T | T1025N (ACC→AAC) | STE6 ← | Confimed |
|  | 0 | 13 | 87053 | (TAT)_36→34_ | intergenic (‑314/+65) | PRE8 ← / ← RPM2 | Not available |
|  | 0.74 | 7 | 128474 | T→A | Y1259* (TAT→TAA) | MDS3 → | Confimed |
|  | 0 | 3 | 151555 | +A | intergenic (‑199/‑1282) | tK(CUU)C ← / → MAK32 | Confirmed |
|  | 0 | 9 | 241053 | (A)_21→22_ | intergenic (‑345/+255) | RNR3 ← / ← FIS1 | Confimed |
|  | 0 | 3 | 286312 | C→T | Q2066* (CAA→TAA) | CDC39 → | Confirmed |
|  | 0 | 7 | 404475 | +G | intergenic (+84/‑995) | MST27 → / → tR(UCU)G1 | Confimed |
|  | 0.86 | 8 | 475932 | Δ1 bp | intergenic (‑154/+67) | tV(CAC)H ← / ← KOG1 | Not available |
|  | 0.49 | 7 | 530034 | A→C | S257S (TCA→TCC) | MTL1 → | Confimed |
|  | 0.65 | 10 | 715141 | A→G | T200T (ACT→ACC) | DAN4 ← | Confirmed |
|  | 0 | 2 | 754982 | C→T | D709N (GAC→AAC) | RIF1 ← | Confimed |
|  | 1 | 13 | 908218 | C→T | S285S (AGC→AGT) | YMR317W → | Not available |
| P11M5 | 0.52 | 8 | 1846 | A→G | T231T (ACT→ACC) | YHL050C ← | - |
|  | 0 | 14 | 12986 | (T)_11→14_ | intergenic (‑110/‑281) | SNO2 ← / → SNZ2 | - |
|  | 0.79 | 1 | 27090 | T→C | E293E (GAA→GAG) | FLO9 ← | - |
|  | 0.83 | 1 | 27105 | A→G | T288T (ACT→ACC) | FLO9 ← | Confirmed |
|  | 0 | 13 | 87053 | (TAT)_36→34_ | intergenic (‑314/+65) | PRE8 ← / ← RPM2 | - |
|  | 0.20 | 7 | 128053 | C→A | S1119* (TCG→TAG) | MDS3 → | - |
|  | 0 | 7 | 400571 | (T)_21→22_ | intergenic (+411/+300) | OLE1 → / ← ERV14 | - |
|  | 0 | 4 | 403520 | (T)_9→10_ | intergenic (‑229/+175) | MPS1 ← / ← MRX9 | - |
|  | 0.49 | 7 | 530034 | A→C | S257S (TCA→TCC) | MTL1 → | - |
|  | 1 | 13 | 908174 | 2 bp→TC | coding (811‑812/3423 nt) | YMR317W → | Not available |
|  | 1 | 13 | 908177 | A→G | I272V (ATT→GTG) | YMR317W → | Not available |
|  | 1 | 13 | 908179 | T→G | I272V (ATT→GTG) | YMR317W → | Not available |
|  | 1 | 13 | 908185 | A→G | S274S (TCA→TCG) | YMR317W → | Not available |
|  | 1 | 13 | 908196 | G→C | W278S (TGG→TCG) | YMR317W → | Not available |
|  | 1 | 13 | 908198 | G→T | A279S (GCA→TCA) | YMR317W → | Not available |
|  | 1 | 13 | 908203 | G→A | T280T (ACG→ACA) | YMR317W → | Not available |
|  | 1 | 13 | 908218 | C→T | S285S (AGC→AGT) | YMR317W → | Not available |
|  | 0.13 | 4 | 958000 | C→A | E114* (GAA→TAA) | YDR248C ← | - |
| P14M2 | 0.29 | 8 | 1846 | A→G | T231T (ACT→ACC) | YHL050C ← | - |
|  | 0 | 1 | 6755 | (A)_19→20_ | intergenic (+4048/+480) | YAL067W‑A → / ← SEO1 | - |
|  | 1 | 1 | 26981 | T→C | S330G (AGC→GGC) | FLO9 ← | Confirmed |
|  | 1 | 1 | 26983 | T→G | N329T (AAC→ACC) | FLO9 ← | Confirmed |
|  | 1 | 1 | 27105 | A→G | T288T (ACT→ACC) | FLO9 ← | Confirmed |
|  | 1 | 2 | 34298 | G→A | E1455K (GAG→AAG) | YBL100W‑B → | Confirmed |
|  | 0 | 13 | 87044 | (TAT)_33→31_ | intergenic (‑305/+74) | PRE8 ← / ← RPM2 | - |
|  | 0 | 13 | 87050 | (TAT)_36→33_ | intergenic (‑311/+65) | PRE8 ← / ← RPM2 | - |
|  | 0 | 13 | 88387 | A→C | L782W (TTG→TGG) | RPM2 ← | - |
|  | 1 | 7 | 128474 | T→A | Y1259* (TAT→TAA) | MDS3 → | Confirmed |
|  | 0 | 7 | 397854 | (A)_20→19_ | intergenic (‑236/‑774) | SDS23 ← / → OLE1 | - |
|  | 0 | 4 | 403520 | (T)_9→10_ | intergenic (‑229/+175) | MPS1 ← / ← MRX9 | - |
|  | 0 | 7 | 404475 | +G | intergenic (+84/‑995) | MST27 → / → tR(UCU)G1 | - |
|  | 0 | 12 | 468701 | T→C | intergenic (‑1832/‑112) | RDN18‑2 ← / → RDN5‑2 | - |
|  | 0 | 10 | 539401 | A→C | intergenic (+135/‑2107) | HIT1 → / → tD(GUC)J4 | - |
|  | 0 | 12 | 612381 | T→G | S5R (AGT→AGG) | THI7 → | - |
|  | 0 | 4 | 930049 | T→C | E103E (GAA→GAG) | RTN1 ← | - |
|  | 0 | 4 | 1489588 | A→T | intergenic (+96/+10) | YDR524W‑C → / ← YDR524C‑B | - |
| P18M3 | 1 | 15 | 92 | Δ3 bp | intergenic (–/‑491) | – / → YOL166W‑A | Not available |
|  | 0.71 | 8 | 1846 | A→G | T231T (ACT→ACC) | YHL050C ← | - |
|  | 0 | 14 | 12986 | (T)_11→14_ | intergenic (‑110/‑281) | SNO2 ← / → SNZ2 | - |
|  | 0.92 | 1 | 26973 | A→G | F332F (TTT→TTC) | FLO9 ← | Confirmed |
|  | 0.92 | 1 | 26981 | T→C | S330G (AGC→GGC) | FLO9 ← | Confirmed |
|  | 0.92 | 1 | 26983 | T→G | N329T (AAC→ACC) | FLO9 ← | Confirmed |
|  | 0.71 | 1 | 27090 | T→C | E293E (GAA→GAG) | FLO9 ← | - |
|  | 0.82 | 1 | 27105 | A→G | T288T (ACT→ACC) | FLO9 ← | Confirmed |
|  | 0 | 4 | 37075 | C→A | G93G (GGC→GGA) | MFG1 → | - |
|  | 0 | 13 | 87053 | (TAT)_36→34_ | intergenic (‑314/+65) | PRE8 ← / ← RPM2 | - |
|  | 0.80 | 7 | 128474 | T→A | Y1259* (TAT→TAA) | MDS3 → | Confirmed |
|  | 0 | 3 | 231024 | (T)_19→20_ | intergenic (+20/‑476) | HCM1 → / → RAD18 | - |
|  | 0 | 4 | 403520 | (T)_9→10_ | intergenic (‑229/+175) | MPS1 ← / ← MRX9 | - |
|  | 0 | 7 | 404475 | +G | intergenic (+84/‑995) | MST27 → / → tR(UCU)G1 | - |
|  | 0.80 | 12 | 468701 | T→C | intergenic (‑1832/‑112) | RDN18‑2 ← / → RDN5‑2 | - |
|  | 0 | 4 | 688076 | (T)_14→15_ | intergenic (+236/‑151) | APC4 → / → VBA4 | - |
|  | 0 | 2 | 723678 | (A)_15→16_ | intergenic (+43/+58) | SRB6 → / ← TRS20 | - |
|  | 0.66 | 13 | 908196 | G→C | W278S (TGG→TCG) | YMR317W → | - |
|  | 0.67 | 13 | 908218 | C→T | S285S (AGC→AGT) | YMR317W → | - |

Table S4: Titer and yield for strains described in Figure 5. Bold: p value <0.05 using two-tailed Student’s t test compared with control.

| **Mutation** | **Titer (mg/L)** | **stdev** | **Yield (mg/g DCW)** | **stdev** |
| --- | --- | --- | --- | --- |
| **Control** | 20.78 | 0.41 | 3.40 | 0.05 |
| **CDC39** | 20.76 | 0.79 | 3.56 | 0.12 |
| **DAN4 T200T** | 20.85 | 0.96 | 3.48 | 0.22 |
| **FLO9 T288T** | 18.70 | 1.35 | 3.16 | 0.24 |
| **tK(CUU)C/MAK32 int** | 19.97 | 2.71 | 3.44 | 0.45 |
| **MDS3** | **17.72** | **1.23** | 2.95 | 0.32 |
| **MST27/tR(UCU)G1 int** | **62.13** | **4.28** | **10.27** | **0.66** |
| **MTL1 S257S** | **13.14** | **1.71** | **2.25** | **0.27** |
| **RIF1 D709N** | 20.59 | 2.10 | 3.37 | 0.32 |
| **RNR3/FIS1 int** | 19.66 | 1.28 | 3.31 | 0.17 |
| **SNO2/SNZ2 int** | 20.75 | 1.10 | 3.58 | 0.27 |
| **STE6 T1025N** | **73.12** | **2.82** | **12.66** | **0.53** |
| **YAL064W‑B/TDA8 int** | 20.29 | 0.43 | 3.35 | 0.16 |
| **YHL050C int** | **19.32** | **0.52** | 3.26 | 0.15 |
| **MST27/tR(UCU)G1 int & STE6 T1025N** | **67.58** | **3.88** | **11.29** | **1.06** |

Table S5: List of plasmids and strains used in study

| **Plasmid** | **Description** |
| --- | --- |
| pQHS1CEN | P_TDH3_-QHS1-T_TDH1_-URA3 (CEN plasmid) |
| pQHS12m | P_TDH3_-QHS1-T_TDH1_-URA3 (2μ plasmid) |
| pFPPCEN | P_THD3_-tHMG1-T_TDH1_-P_CCW12_-HMG2(K6R)-T_ENO2_-P_PGK1_-UPC2-1-T_PGK1_-P_HHF2_-ERG20-T_ADH3_ -LEU2 (CEN Plasmid) |
| pFPP2m | P_THD3_-tHMG1-T_TDH1_-P_CCW12_-HMG2(K6R)-T_ENO2_-P_PGK1_-UPC2-1-T_PGK1_-P_HHF2_-ERG20-T_ADH3_ -LEU2 (2μ Plasmid) |
| pQHS12mH | P_TDH3_-QHS1-T_TDH1_-HIS3 (2μ plasmid) |
| pSTE6 | P_TDH3_-STE6-T_TDH1_-URA3 (2μ plasmid) |
| pSTE6m | P_TDH3_-STE6 T1025N-T_TDH1_-URA3 (2μ plasmid) |
| **Strains** | **Genotype** |
| BY4741 | *MATa his3Δ1 leu2Δ0 met15Δ0 ura3Δ0* |
| YAG101 | BY4741 ura3::P_TDH3_-QHS1-T_TDH1_::URA3 |
| YAG102 | BY4741 /pQHS1CEN |
| YAG103 | BY4741 /pQHS12m |
| YAG104 | BY4741 leu2:: P_THD3_-tHMG1-T_TDH1_-P_CCW12_-HMG2(K6R)-T_ENO2_-P_PGK1-_UPC2-1-T_PGK1_-P_HHF2_-ERG20-T_ADH1_::LEU2 |
| YAG105 | BY4741 ura3:: P_TDH3_-QHS1-T_TDH1_::URA3 leu2:: P_THD3_-tHMG1-T_TDH1_-P_CCW12_-HMG2(K6R)-T_ENO2_-P_PGK1-_UPC2-1-T_PGK1_-P_HHF2_-ERG20-T_ADH2_::LEU2 |
| YAG106 | BY4741 ura3:: P_TDH3_-QHS1-T_TDH1_::URA3 /pFPPCEN |
| YAG107 | BY4741 ura3:: P_TDH—_QHS1-T_TDH1_::URA3 /pFPP2m |
| YAG108 | BY4741 leu2:: P_THD3_-tHMG1-T_TDH1_-P_CCW12_-HMG2(K6R)-T_ENO2_-P_PGK1-_UPC2-1-T_PGK1_-P_HHF2_-ERG20-T_ADH3_::LEU2 /pQHS12m |
| YAG109 | BY4741 /pQHS12m /pFPP2m |
| YAG110 | BY4741 *ctt1*Δ |
| YAG111 | BY4741 *ctt1*Δ ura3:: P_TDH3_-QHS1-T_TDH1_ |
| YAG112 | BY4741 *ctt1*Δ /pQHS1CEN |
| YAG113 | BY4741 *ctt1*Δ /pQHS12m |
| YAG114 | BY4741 *ctt1*Δ leu2::P_THD3_-tHMG1-T_TDH1_-P_CCW12_-HMG2(K6R)-T_ENO2_-P_PGK1-_UPC2-1-T_PGK1_-P_HHF2_-ERG20-T_ADH1_::LEU2 |
| YAG115 | BY4741 *ctt1*Δ ura3::P_TDH3_-QHS1-T_TDH1_::URA3 leu2::P_THD3_-tHMG1-T_TDH1_-P_CCW12_-HMG2(K6R)-T_ENO2_-P_PGK1-_UPC2-1-T_PGK1_-P_HHF2_-ERG20-T_ADH2_::LEU2 |
| YAG116 | YAG115 /pQHS12mH |
| YAG117 | Population 11 mutant 1 /pQHS12mH |
| YAG118 | BY4741 *ctt1*Δ his3:: P_TDH3_-QHS1-T_TDH1_::HIS3 leu2::P_THD3_-tHMG1-T_TDH1_-P_CCW12_-HMG2(K6R)-T_ENO2_-P_PGK1-_UPC2-1-T_PGK1_-P_HHF2_-ERG20-T_ADH2_::LEU2 |
| YAG119 | BY4741 *ctt1*Δ leu2:: P_THD3_-tHMG1-T_TDH1_-P_CCW12_-HMG2(K6R)-T_ENO2_-P_PGK1-_UPC2-1-T_PGK1_-P_HHF2_-ERG20-T_ADH1_::LEU2_,_ CDC39 |
| YAG120 | BY4741 *ctt1*Δ leu2:: P_THD3_-tHMG1-T_TDH1_-P_CCW12_-HMG2(K6R)-T_ENO2_-P_PGK1-_UPC2-1-T_PGK1_-P_HHF2_-ERG20-T_ADH1_::LEU2_,_ DAN4 T200T |
| YAG121 | BY4741 *ctt1*Δ leu2:: P_THD3_-tHMG1-T_TDH1_-P_CCW12_-HMG2(K6R)-T_ENO2_-P_PGK1-_UPC2-1-T_PGK1_-P_HHF2_-ERG20-T_ADH1_::LEU2_,_ FLO9 T288T |
| YAG122 | BY4741 *ctt1*Δ leu2:: P_THD3_-tHMG1-T_TDH1_-P_CCW12_-HMG2(K6R)-T_ENO2_-P_PGK1-_UPC2-1-T_PGK1_-P_HHF2_-ERG20-T_ADH1_::LEU2_,_ tK(CUU)C/MAK32 int |
| YAG123 | BY4741 *ctt1*Δ leu2:: P_THD3_-tHMG1-T_TDH1_-P_CCW12_-HMG2(K6R)-T_ENO2_-P_PGK1-_UPC2-1-T_PGK1_-P_HHF2_-ERG20-T_ADH1_::LEU2_,_ MDS3 |
| YAG124 | BY4741 *ctt1*Δ leu2:: P_THD3_-tHMG1-T_TDH1_-P_CCW12_-HMG2(K6R)-T_ENO2_-P_PGK1-_UPC2-1-T_PGK1_-P_HHF2_-ERG20-T_ADH1_::LEU2_,_ MST27/tR(UCU)G1 int |
| YAG125 | BY4741 *ctt1*Δ leu2:: P_THD3_-tHMG1-T_TDH1_-P_CCW12_-HMG2(K6R)-T_ENO2_-P_PGK1-_UPC2-1-T_PGK1_-P_HHF2_-ERG20-T_ADH1_::LEU2_,_ MTL1 S257S |
| YAG126 | BY4741 *ctt1*Δ leu2:: P_THD3_-tHMG1-T_TDH1_-P_CCW12_-HMG2(K6R)-T_ENO2_-P_PGK1-_UPC2-1-T_PGK1_-P_HHF2_-ERG20-T_ADH1_::LEU2_,_ RIF1 D709N |
| YAG127 | BY4741 *ctt1*Δ leu2:: P_THD3_-tHMG1-T_TDH1_-P_CCW12_-HMG2(K6R)-T_ENO2_-P_PGK1-_UPC2-1-T_PGK1_-P_HHF2_-ERG20-T_ADH1_::LEU2_,_ RNR3/FIS1 int |
| YAG128 | BY4741 *ctt1*Δ leu2:: P_THD3_-tHMG1-T_TDH1_-P_CCW12_-HMG2(K6R)-T_ENO2_-P_PGK1-_UPC2-1-T_PGK1_-P_HHF2_-ERG20-T_ADH1_::LEU2_,_ SNO2/SNZ2 int |
| YAG129 | BY4741 *ctt1*Δ leu2:: P_THD3_-tHMG1-T_TDH1_-P_CCW12_-HMG2(K6R)-T_ENO2_-P_PGK1-_UPC2-1-T_PGK1_-P_HHF2_-ERG20-T_ADH1_::LEU2_,_ STE6 T1025N |
| YAG130 | BY4741 *ctt1*Δ leu2:: P_THD3_-tHMG1-T_TDH1_-P_CCW12_-HMG2(K6R)-T_ENO2_-P_PGK1-_UPC2-1-T_PGK1_-P_HHF2_-ERG20-T_ADH1_::LEU2_,_ YAL064W‑B/TDA8 int |
| YAG131 | BY4741 *ctt1*Δ leu2:: P_THD3_-tHMG1-T_TDH1_-P_CCW12_-HMG2(K6R)-T_ENO2_-P_PGK1-_UPC2-1-T_PGK1_-P_HHF2_-ERG20-T_ADH1_::LEU2_,_ YHL050C int |
| YAG132 | BY4741 *ctt1*Δ his3:: P_TDH3_-QHS1-T_TDH1_::HIS3 leu2:: P_THD3_-tHMG1-T_TDH1_-P_CCW12_-HMG2(K6R)-T_ENO2_-P_PGK1-_UPC2-1-T_PGK1_-P_HHF2_-ERG20-T_ADH1_::LEU2_,_ CDC39 |
| YAG133 | BY4741 *ctt1*Δ his3:: P_TDH3_-QHS1-T_TDH1_::HIS3 leu2:: P_THD3_-tHMG1-T_TDH1_-P_CCW12_-HMG2(K6R)-T_ENO2_-P_PGK1-_UPC2-1-T_PGK1_-P_HHF2_-ERG20-T_ADH1_::LEU2_,_ DAN4 T200T |
| YAG134 | BY4741 *ctt1*Δ his3:: P_TDH3_-QHS1-T_TDH1_::HIS3 leu2:: P_THD3_-tHMG1-T_TDH1_-P_CCW12_-HMG2(K6R)-T_ENO2_-P_PGK1-_UPC2-1-T_PGK1_-P_HHF2_-ERG20-T_ADH1_::LEU2_,_ FLO9 T288T |
| YAG135 | BY4741 *ctt1*Δ his3:: P_TDH3_-QHS1-T_TDH1_::HIS3 leu2:: P_THD3_-tHMG1-T_TDH1_-P_CCW12_-HMG2(K6R)-T_ENO2_-P_PGK1-_UPC2-1-T_PGK1_-P_HHF2_-ERG20-T_ADH1_::LEU2_,_ tK(CUU)C/MAK32 int |
| YAG136 | BY4741 *ctt1*Δ his3:: P_TDH3_-QHS1-T_TDH1_::HIS3 leu2:: P_THD3_-tHMG1-T_TDH1_-P_CCW12_-HMG2(K6R)-T_ENO2_-P_PGK1-_UPC2-1-T_PGK1_-P_HHF2_-ERG20-T_ADH1_::LEU2_,_ MDS3 |
| YAG137 | BY4741 *ctt1*Δ his3:: P_TDH3_-QHS1-T_TDH1_::HIS3 leu2:: P_THD3_-tHMG1-T_TDH1_-P_CCW12_-HMG2(K6R)-T_ENO2_-P_PGK1-_UPC2-1-T_PGK1_-P_HHF2_-ERG20-T_ADH1_::LEU2_,_ MST27/tR(UCU)G1 int |
| YAG138 | BY4741 *ctt1*Δ his3:: P_TDH3_-QHS1-T_TDH1_::HIS3 leu2:: P_THD3_-tHMG1-T_TDH1_-P_CCW12_-HMG2(K6R)-T_ENO2_-P_PGK1-_UPC2-1-T_PGK1_-P_HHF2_-ERG20-T_ADH1_::LEU2_,_ MTL1 S257S |
| YAG139 | BY4741 *ctt1*Δ his3:: P_TDH3_-QHS1-T_TDH1_::HIS3 leu2:: P_THD3_-tHMG1-T_TDH1_-P_CCW12_-HMG2(K6R)-T_ENO2_-P_PGK1-_UPC2-1-T_PGK1_-P_HHF2_-ERG20-T_ADH1_::LEU2_,_ RIF1 D709N |
| YAG140 | BY4741 *ctt1*Δ his3:: P_TDH3_-QHS1-T_TDH1_::HIS3 leu2:: P_THD3_-tHMG1-T_TDH1_-P_CCW12_-HMG2(K6R)-T_ENO2_-P_PGK1-_UPC2-1-T_PGK1_-P_HHF2_-ERG20-T_ADH1_::LEU2_,_ RNR3/FIS1 int |
| YAG141 | BY4741 *ctt1*Δ his3:: P_TDH3_-QHS1-T_TDH1_::HIS3 leu2:: P_THD3_-tHMG1-T_TDH1_-P_CCW12_-HMG2(K6R)-T_ENO2_-P_PGK1-_UPC2-1-T_PGK1_-P_HHF2_-ERG20-T_ADH1_::LEU2_,_ SNO2/SNZ2 int |
| YAG142 | BY4741 *ctt1*Δ his3:: P_TDH3_-QHS1-T_TDH1_::HIS3 leu2:: P_THD3_-tHMG1-T_TDH1_-P_CCW12_-HMG2(K6R)-T_ENO2_-P_PGK1-_UPC2-1-T_PGK1_-P_HHF2_-ERG20-T_ADH1_::LEU2_,_ STE6 T1025N |
| YAG143 | BY4741 *ctt1*Δ his3:: P_TDH3_-QHS1-T_TDH1_::HIS3 leu2:: P_THD3_-tHMG1-T_TDH1_-P_CCW12_-HMG2(K6R)-T_ENO2_-P_PGK1-_UPC2-1-T_PGK1_-P_HHF2_-ERG20-T_ADH1_::LEU2_,_ YAL064W‑B/TDA8 int |
| YAG144 | BY4741 *ctt1*Δ his3:: P_TDH3_-QHS1-T_TDH1_::HIS3 leu2:: P_THD3_-tHMG1-T_TDH1_-P_CCW12_-HMG2(K6R)-T_ENO2_-P_PGK1-_UPC2-1-T_PGK1_-P_HHF2_-ERG20-T_ADH1_::LEU2_,_ YHL050C int |
| YAG145 | BY4741 *ctt1*Δ his3:: P_TDH3_-QHS1-T_TDH1_::HIS3 leu2:: P_THD3_-tHMG1-T_TDH1_-P_CCW12_-HMG2(K6R)-T_ENO2_-P_PGK1-_UPC2-1-T_PGK1_-P_HHF2_-ERG20-T_ADH1_::LEU2_,_ STE6 T1025N, MST27/tR(UCU)G1 int |
| YAG146 | BY4741 *ctt1*Δ his3:: P_TDH3_-QHS1-T_TDH1_::HIS3 leu2:: P_THD3_-tHMG1-T_TDH1_-P_CCW12_-HMG2(K6R)-T_ENO2_-P_PGK1-_UPC2-1-T_PGK1_-P_HHF2_-ERG20-T_ADH1_::LEU2_,_ ste6∆ |
| YAG147 | BY4741 *ctt1*Δ his3:: P_TDH3_-QHS1-T_TDH1_::HIS3 leu2:: P_THD3_-tHMG1-T_TDH1_-P_CCW12_-HMG2(K6R)-T_ENO2_-P_PGK1-_UPC2-1-T_PGK1_-P_HHF2_-ERG20-T_ADH1_::LEU2_,_ /pSTE6 |
| YAG148 | BY4741 *ctt1*Δ his3:: P_TDH3_-QHS1-T_TDH1_::HIS3 leu2:: P_THD3_-tHMG1-T_TDH1_-P_CCW12_-HMG2(K6R)-T_ENO2_-P_PGK1-_UPC2-1-T_PGK1_-P_HHF2_-ERG20-T_ADH1_::LEU2_,_ /pSTE6m |
| YAG149 | BY4741 *ctt1*Δ his3:: P_TDH3_-ZSS1-T_TDH1_::HIS3 leu2::P_THD3_-tHMG1-T_TDH1_-O_CCW12_-HMG2(K6R)-T_ENO2_-P_PGK1-_UPC2-1-T_PGK1_-P_HHF2_-ERG20-T_ADH1_::LEU2 |
| YAG150 | BY4741 *ctt1*Δ his3:: P_TDH3_-ZSS1-T_TDH1_::HIS3 leu2::P_THD3_-tHMG1-T_TDH1_-O_CCW12_-HMG2(K6R)-T_ENO2_-P_PGK1-_UPC2-1-T_PGK1_-P_HHF2_-ERG20-T_ADH1_::LEU2_,_ /pSTE6m |

Table S6: Donor sequence for reconstruction of mutations

| **Mutation** | **Donor sequence** |
| --- | --- |
| **CDC39** | CTTTGGTCTCACCAAAACCTAATTGTTGTAAAATTAGTTTAATTTCGGGAACACTTTATACAAAGGGCAGGTCTAAAAGATTAATATCGTTATTGTTTATCAACTGAGTAAAGAAAACGAACACTTTGTACAAAGGGCGTTTTAGAGAGAGACCTTTC |
| **DAN4 T200T** | CTTTGGTCTCACCAAAACGTGGTAGAAGTAGTAGAGGTTGTAGGAGTAGTCGATGTGGTAGAGGTTGTAGGAGTGGTAGAAGTAGTAGAGGTTGTAGGAGTAGTCGATGTGGTAGAAGGTCGATGTGGTAGAAGTTGTGTTTTAGAGAGAGACCTTTC |
| **FLO9 T288T** | CTTTGGTCTCACCAAAACGTTGGTACCGGTGACGGTGGTCATTTCAGTAGATGTAGAGGTGAAAGTACCGGTCCATGGTTCCGTTGTAGTTATGGTAGTACTGACAGTATAATTTGAAAGATGTAGAAGTGAAAGTACGTTTTAGAGAGAGACCTTTC |
| **tK(CUU)C/MAK32 int** | CTTTGGTCTCACCAAAACCCGTATATGATAATATATTGATAATATAACTATTAGTTGATAGACGATAGTGGATTTTTATTCCAACAATTCTATATACGTAAAATTATAGCCTTTACCACTATAGTTGATAGACGATAGGTTTTAGAGAGAGACCTTTC |
| **MDS3** | CTTTGGTCTCACCAAAACTGTTAGTGCTACTGCTGCTGATGGAACCTTAATTCATATCAAACAAATGAAGATTAAAACTTCCGATGTTGGTTTGTTGATCATCGTTATCATCAAAACTAATTCATATCAAACAAATGAGTTTTAGAGAGAGACCTTTC |
| **MST27/tR(UCU)G1 int** | CTTTGGTCTCACCAAAACAAATGGGTCCAACACGAATCGACTTTTCGAGGCTTCCTTCGGCCGTTTTCGGGCCAGTTAGTGCTGATTATATATCATACTCTAGTTTATGTTCGCTTTAAGGCTTCTTCGGCCGTTTTCGTTTTAGAGAGAGACCTTTC |
| **MTL1 S257S** | CTTTGGTCTCACCAAAACTGTGGAGAGGGTGAAATATGATGATGATGATGATGATGAGGATGAGGATGAGGATGATGATGAGGATGATGAGGATGAGGATGATGAAAATGAGGAGGAGTGATGATGATGATGAGGATGGTTTTAGAGAGAGACCTTTC |
| **RIF1 D709N** | CTTTGGTCTCACCAAAACGATGATCTGCGGGGGCAGGCTTGAAATGTTTTTTAACTTTACACCTTCTGAAGCTATAACACTCATTGGGTGAATATGCTTTTTATATTTTCTTTCCAATTTTTAACTTTACACCTTCTGGTTTTAGAGAGAGACCTTTC |
| **RNR3/FIS1 int** | CTTTGGTCTCACCAAAACCAACATTGCGTGCCGTTGTTCTTTTGTTTTTTTTTTTTTTTTTTTTTTCGTTGTTGTCGCAGCAACGACACCTAGGCGCTGCTCAAAGGGGCAAAAACCCTTTTTTTTTTTTTTTTTTCGGTTTTAGAGAGAGACCTTTC |
| **SNO2/SNZ2 int** | CTTTGGTCTCACCAAAACCAGCAACCGGGGTCATGGTAAGTGTGCTTGCTACTCAAACAGAAAAAAATGGTCTTTTATCAATGAATAACTTTTTTTTTTTGTAGCTGAATGATGACTACTCAAACAGAAAAAAATGTTTTAGAGAGAGACCTTTC |
| **STE6 T1025N** | CTTTGGTCTCACCAAAACTGCTGCCAGTTGGATCTATAGGATTCTTGATGAAAAGCATAATAACCTAGAGGTTGAAAACAATAATGCTAGAACAGTGGGAATAGCTGGTCACACCTACTGAAAAGCATAATACCCTAGGTTTTAGAGAGAGACCTTTC |
| **YAL064W‑B/TDA8 int** | CTTTGGTCTCACCAAAACGGTGGTAATGATGAAGTAATTTCCTGACTTGTTGTTGTACTGGTAACAGGGGGTAATGATGAAGTAATTTCCTGACTTGTTGTTGCACTGGTAACAGGTGGTTGTTGTACTGGTAACAGGGTTTTAGAGAGAGACCTTTC |
| **YHL050C int** | CTTTGGTCTCACCAAAACAAGAGATGGGGGGGGGGGGCTCTGTTATCTATTATCTAGAAAAAACAGTCGGGCCGCAAGGAATCGTAAGGGTGAATTGCCACCAATTAAGGAAGGCTGTATTATCTAGAAAAAACAGTTGTTTTAGAGAGAGACCTTTC |

**List of Additional Figures**


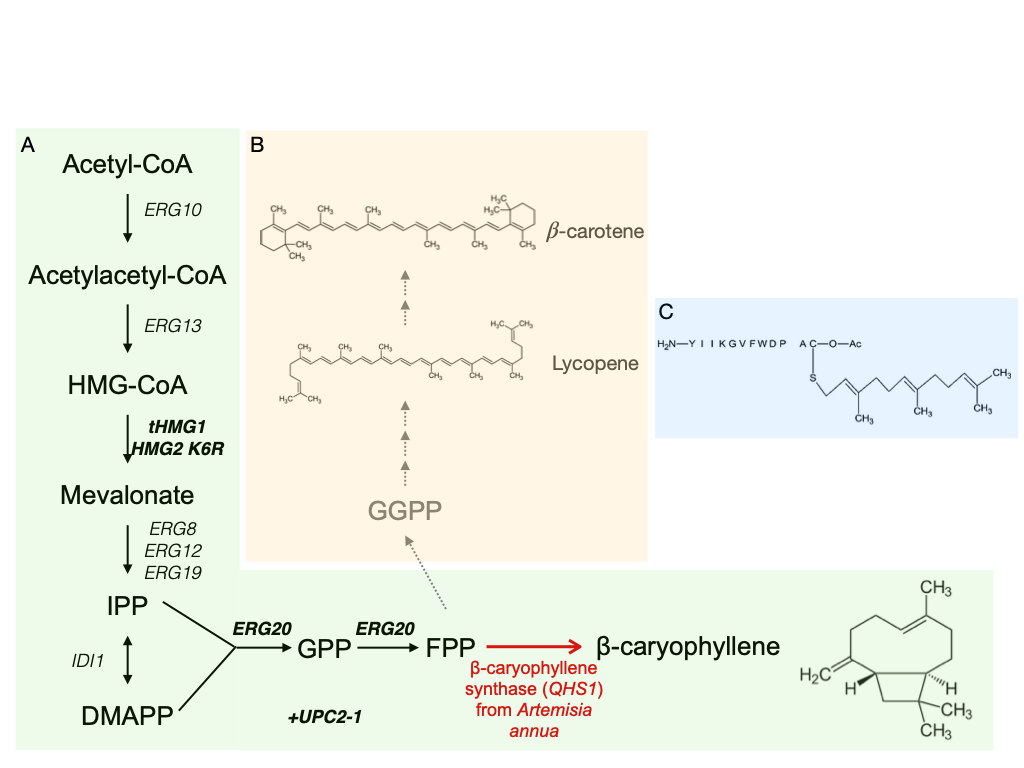


Figure S1: Biosynthesis pathway for and structure of beta-caryophyllene. A) Biosynthesis of beta-caryophylene starting from acetyl-CoA. Bolded genes are overexpressed in the FPP overproduction cassette. QHS1 is the codon optimized beta-caryophyllene synthase from Artemisia annua. B) Relation of beta-caryophyllene to other commonly targeted carotenoids lycopene and beta-carotene. C) Structure of a-factor, the target for Ste6.

Figure S2: Antioxidant potential of β-caryophyllene, 3 biologic replicates for each strain were exposed to various hydrogen peroxide concentrations, and spot assay were done with serial 1/10X dilutions.

**
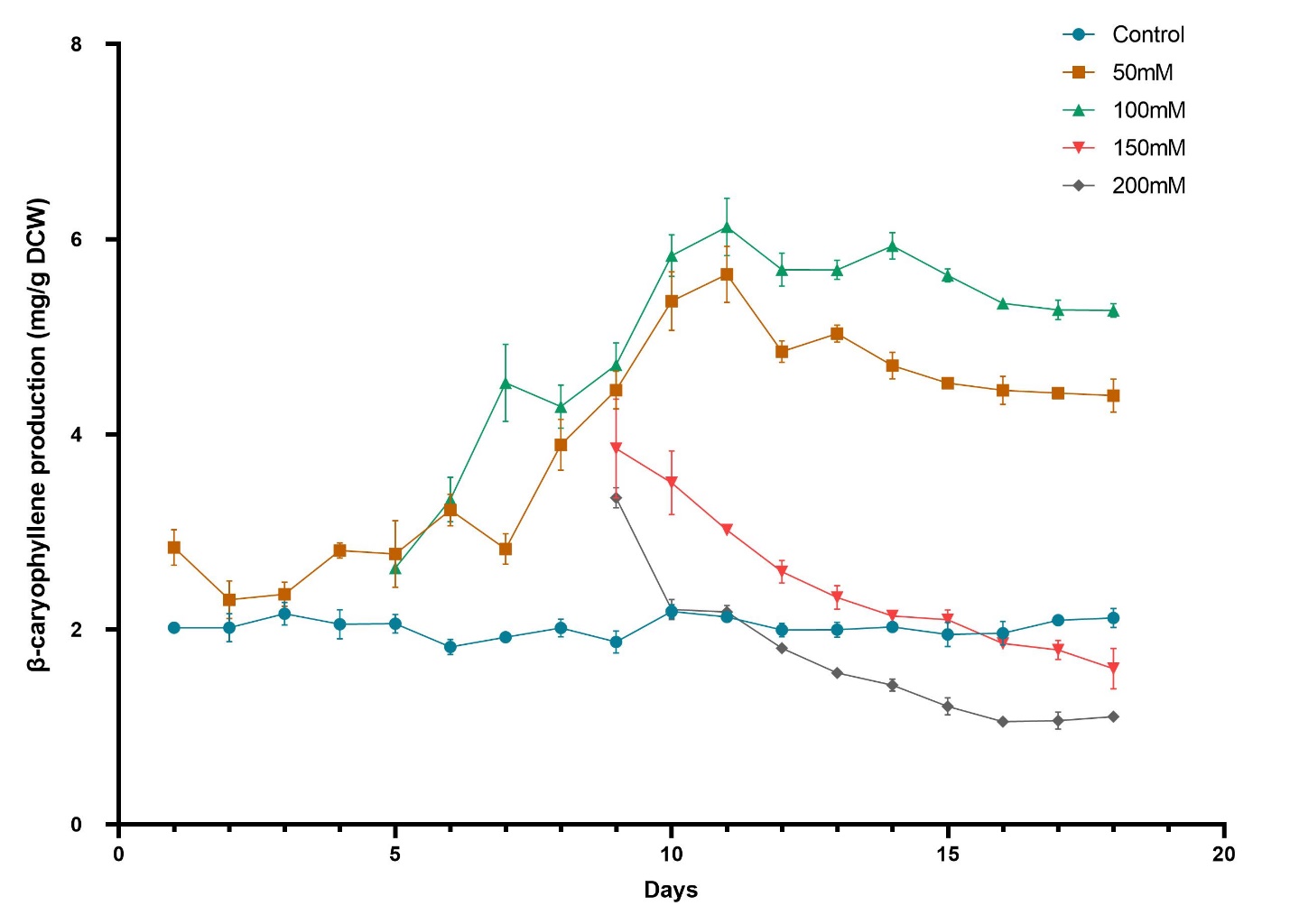
**

Figure S3: Production observed during evolution experiments. Production was measured in 20 ml test tube with 3 ml culture overlayed with 500 μl dodecane. 200 μl of the culture was transferred daily from the previous culture to inoculate in fresh media after oxidative stress challenge (if any). 100 μl of the dodecane layer is sampled for β-caryophyllene quantification.

Figure S4: Spot assays for parental strain as well as the best population sample from evolution experiment. Cells were exposed to different hydrogen peroxide concentrations for 30 min and spotted.

10^-2^

10^-3^

10^-4^

10^-5^

Control

500mM

1M

Dilution

YAG115


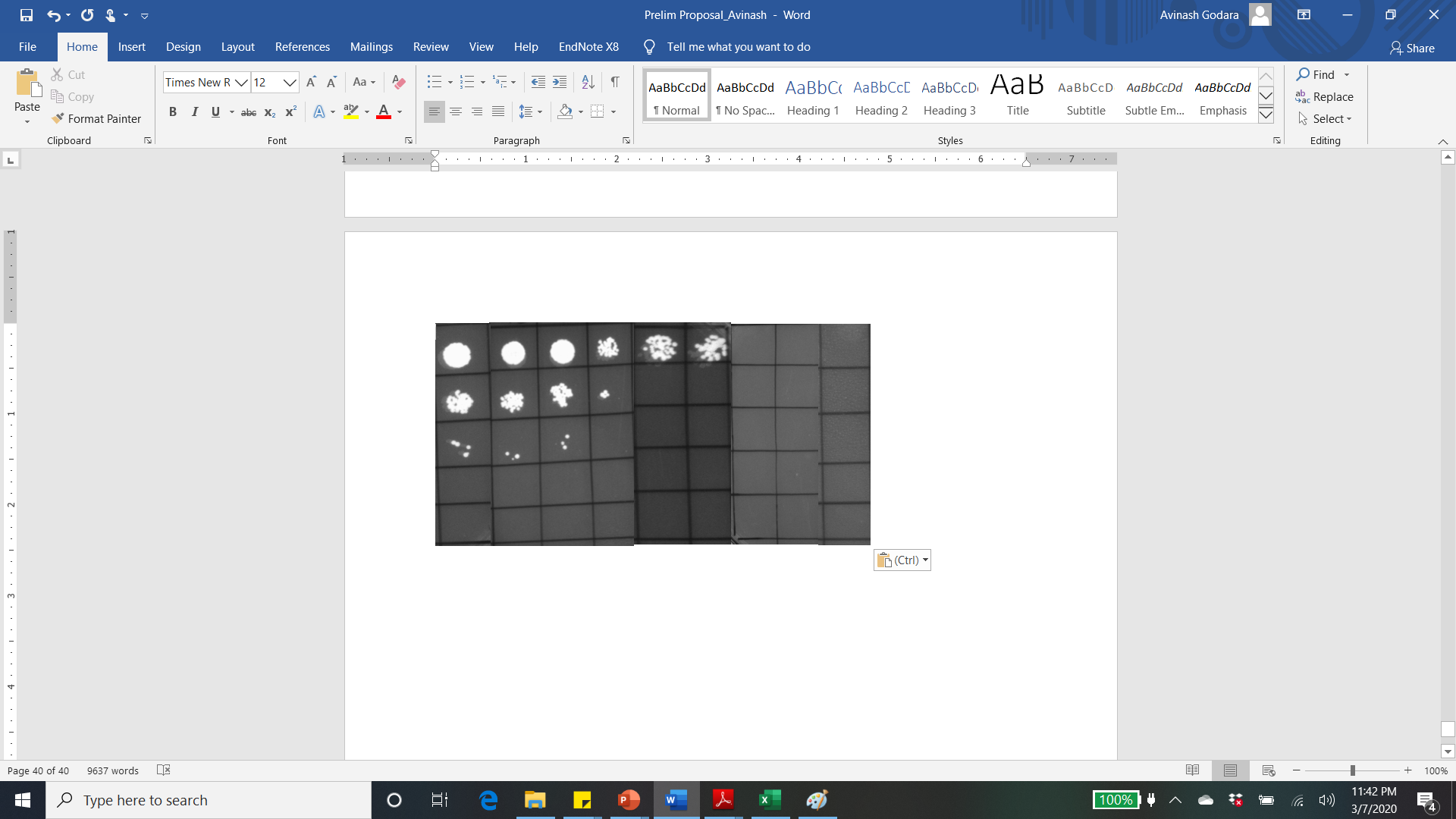


Strain

10^-1^

10^-2^

10^-3^

10^-4^

10^-5^

Control

500mM

1M

Dilution


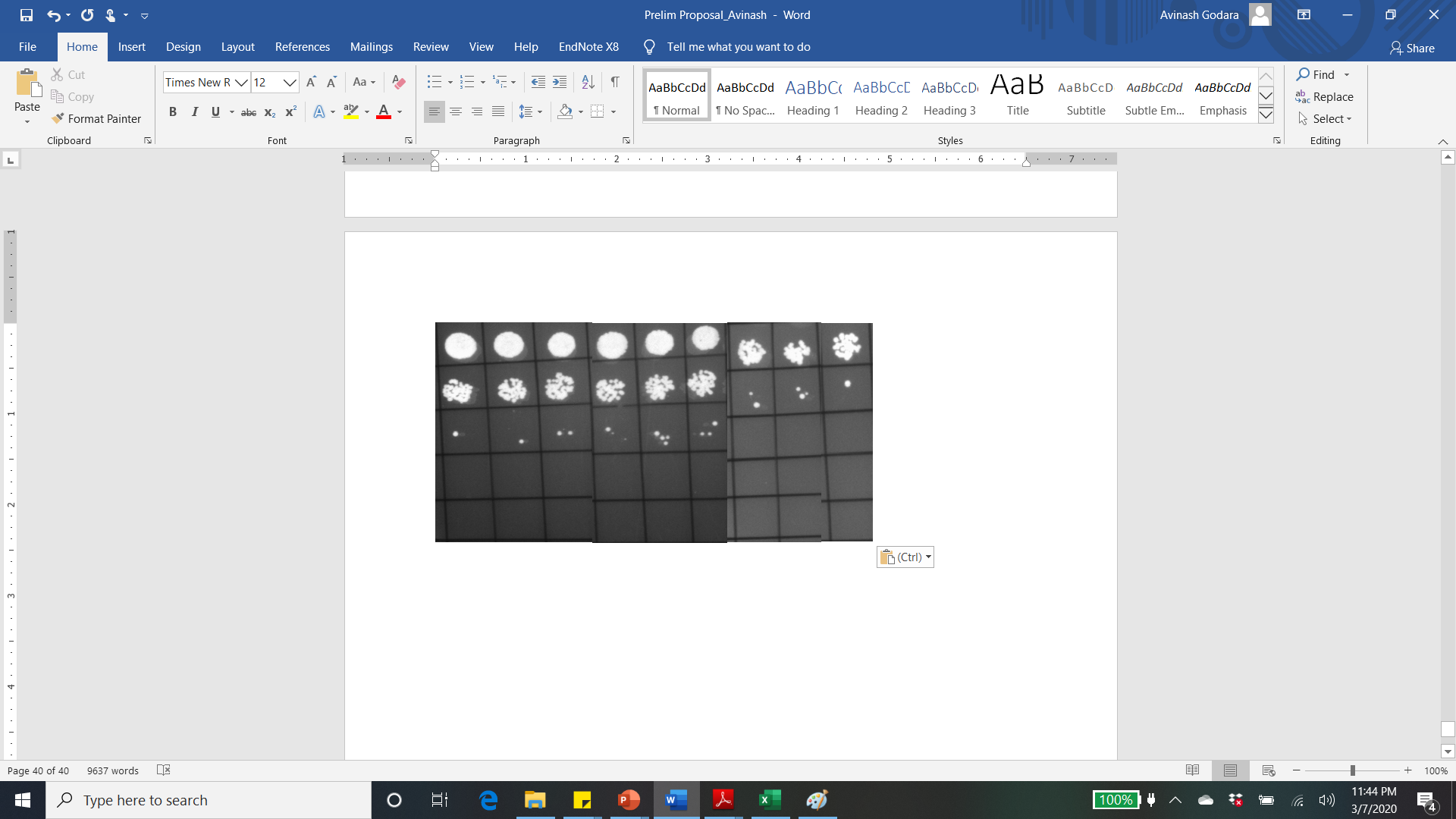


P2 day 11


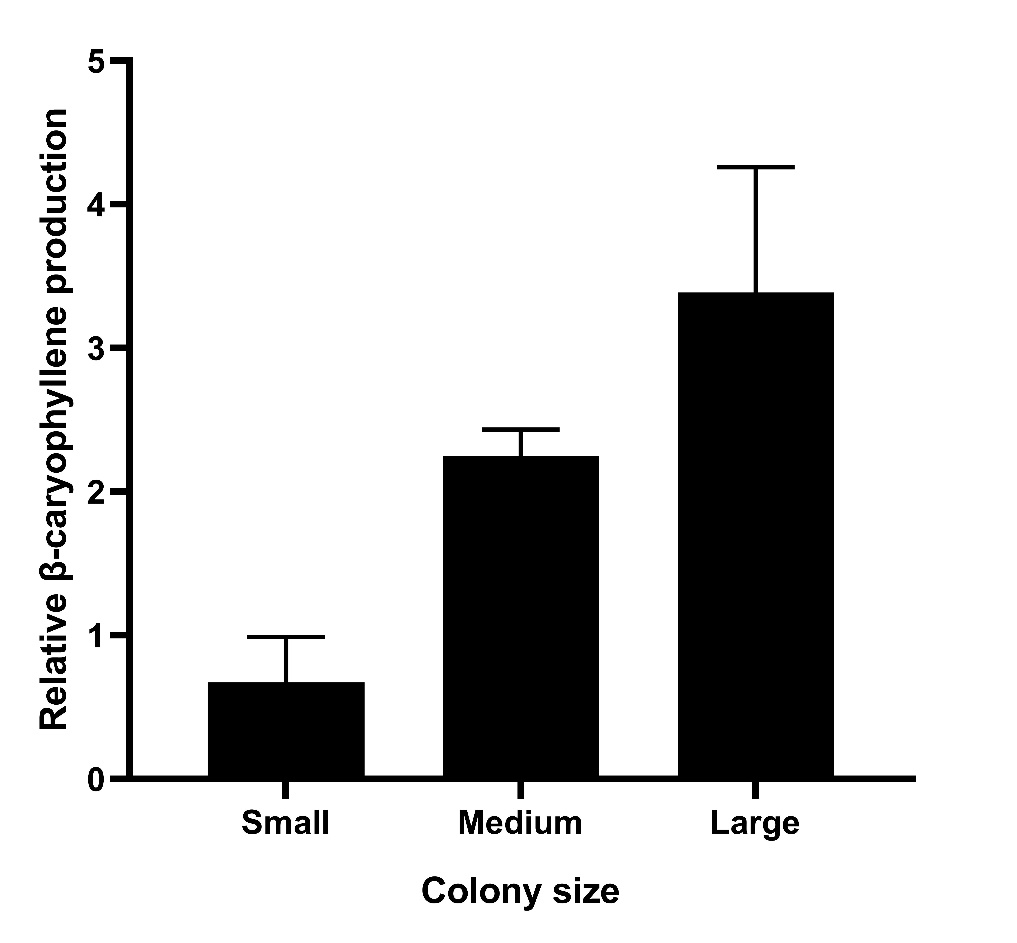


Figure S5: Relative caryophyllene production between average of 3 colonies picked of various sizes isolated from screening. Production was measured in 20 ml test tube with 3 ml culture overlayed with 500 μl dodecane.


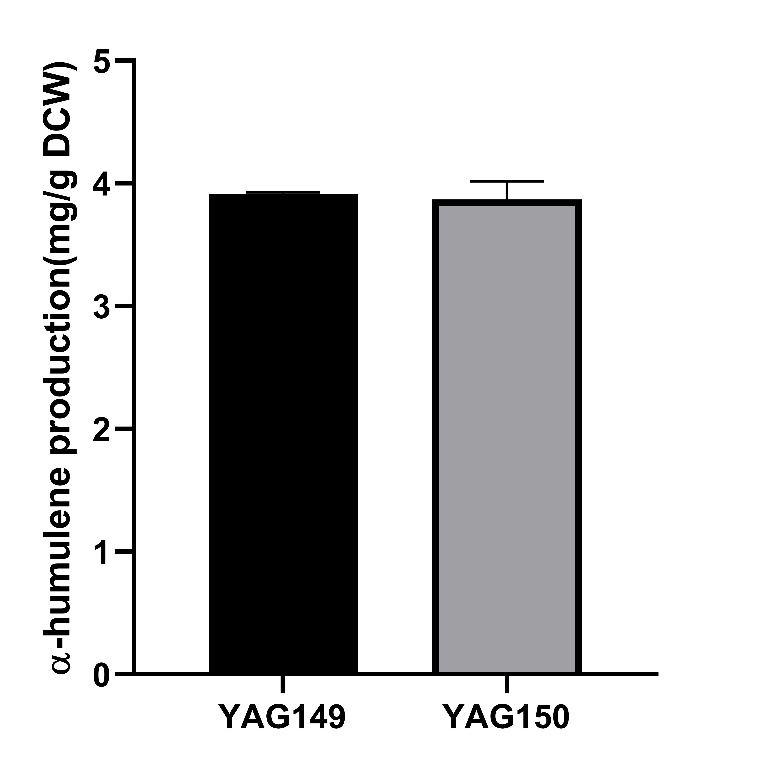


Figure S6: alpha humulene production under overexpressed *STE6 T1025N.* Production was measured in 20 ml test tube with 3 ml culture overlayed with 500 μl dodecane.
